# Dynamin-2 mutations linked to Centronuclear Myopathy impair actin-dependent trafficking in muscle cells

**DOI:** 10.1038/s41598-017-04418-w

**Published:** 2017-07-04

**Authors:** Arlek M. González-Jamett, Ximena Baez-Matus, María José Olivares, Fernando Hinostroza, Maria José Guerra-Fernández, Jacqueline Vasquez-Navarrete, Mai Thao Bui, Pascale Guicheney, Norma Beatriz Romero, Jorge A. Bevilacqua, Marc Bitoun, Pablo Caviedes, Ana M. Cárdenas

**Affiliations:** 1 Centro Interdisciplinario de Neurociencia de Valparaíso. Facultad de Ciencias, Universidad de Valparaíso, Valparaíso, Chile; 2 Université Sorbonne, UPMC Univ Paris 06, INSERM UMRS974, CNRS FRE3617, Center for Research in Myology, Paris, France; 3 Centre de référence de Pathologie Neuromusculaire Paris-Est, Institut de Myologie, GHU Pitié-Salpêtrière, Assistance Publique-Hôpitaux de Paris, GH Pitié-Salpêtrière, Paris, France; 4 INSERM, UMR_S1166, Sorbonne Universités, UPMC Univ Paris 06, UMR_S1166, Institute of Cardiometabolism and Nutrition (ICAN), Paris, France; 5 Programa de Anatomía y Biología del Desarrollo, ICBM, Facultad de Medicina, Departamento de Neurología y Neurocirugía, Hospital Clínico Universidad de Chile, Universidad de Chile, Santiago, Chile; 6 Research Center for Myology, UPMC Univ Paris 06 and INSERM UMRS 974, Institute of Myology, Paris, France; 7 Programa de Farmacología Molecular y Clinica, ICBM, Facultad de Medicina, Universidad de Chile, Santiago, Chile; 8 Doctorado en Ciencias, mención Neurociencia, Universidad de Valparaíso, Valparaíso, Chile

## Abstract

Dynamin-2 is a ubiquitously expressed GTP-ase that mediates membrane remodeling. Recent findings indicate that dynamin-2 also regulates actin dynamics. Mutations in dynamin-2 cause dominant centronuclear myopathy (CNM), a congenital myopathy characterized by progressive weakness and atrophy of skeletal muscles. However, the muscle-specific roles of dynamin-2 affected by these mutations remain elusive. Here we show that, in muscle cells, the GTP-ase activity of dynamin-2 is involved in *de novo* actin polymerization as well as in actin-mediated trafficking of the glucose transporter GLUT4. Expression of dynamin-2 constructs carrying CNM-linked mutations disrupted the formation of new actin filaments as well as the stimulus-induced translocation of GLUT4 to the plasma membrane. Similarly, mature muscle fibers isolated from heterozygous knock-in mice that harbor the dynamin-2 mutation p.R465W, an animal model of CNM, exhibited altered actin organization, reduced actin polymerization and impaired insulin-induced translocation of GLUT4 to the sarcolemma. Moreover, GLUT4 displayed aberrant perinuclear accumulation in biopsies from CNM patients carrying dynamin-2 mutations, further suggesting trafficking defects. These results suggest that dynamin-2 is a key regulator of actin dynamics and GLUT4 trafficking in muscle cells. Our findings also support a model in which impairment of actin-dependent trafficking contributes to the pathological mechanism in dynamin-2-associated CNM.

## Introduction

Dynamins are mechano-chemical large GTP-ases, whose catalytic activity is required in several membrane-based processes including endocytosis, vesicle trafficking, and exocytosis^1–4^. These proteins also exhibit a critical role in actin cytoskeleton dynamics by promoting elongation^5^, remodeling^6^ and stabilizing actin filaments^7^.

Dynamins are composed of five conserved domains: an N-terminal GTP-ase domain, a middle structural domain, a pleckstrin homology (PH) domain that binds phosphoinositides, a GTP-ase effector domain (GED), and a C-terminal proline/arginine-rich domain (PRD) that binds SH3-domain-containing partners^1–4^. Three dynamin isoforms have been described in mammals, which share approximately 80% of sequence homology and exhibit different expression patterns: dynamin-1 is expressed in neuronal cells, dynamin-2 is ubiquitously expressed, and dynamin-3 is localized in brain, heart, testis and lungs^1–4^. Mutations mainly clustered into the middle and PH domains of dynamin-2 cause autosomal dominant centronuclear myopathy (CNM), a rare congenital myopathy manifested by myalgia, fatigability and progressive weakness and atrophy of skeletal muscles^8^. Despite the ubiquitous expression of dynamin-2, CNM–linked mutations induce a tissue specific disease^9, 10^ suggesting that specific functions of dynamin-2 in skeletal muscles are affected in the course of CNM.

The role of dynamin-2 in muscle tissues is not fully understood. Reportedly, it localizes in the I-band centered on the Z-line^11, 12^ and participates in transverse tubule (T-tubules) organization^13^. Congruently, the specific ablation of dynamin-2 modifies the organization and size of myofibers in mice^13^ and *zebrafish* ^14^ and dramatically decreases the muscle mass in mice^13^. Remarkably, CNM-associated dynamin-2 mutations have been shown to cause sarcomere and neuromuscular junction disorganization in mice^13^ and *zebrafish* skeletal muscles^14, 15^, as well as T-tubule fragmentation in *Drosophila melanogaster* muscles^16^. Formation and maintenance of such muscle structures requires abundant intracellular membrane trafficking^17^. Hence, impairment in these cellular processes could underlie the structural alterations observed in dynamin-2-associated CNM.

Actin cytoskeleton is an important regulator of intracellular trafficking^18, 19^. Three actin isoforms exist in skeletal muscle: alpha-actin that mediates sarcomeric organization and muscle contraction^20^ and cytoskeletal beta- and gamma-actins^20^ that regulate trafficking and stability of plasma membrane proteins such as nicotinic receptors^21^, Ca^2+^ channels^22^ and the glucose transporter GLUT4^23, 24^. Similar to that reported for dynamin-2^11, 12^, cytoskeletal gamma-actin localizes in areas of active membrane remodeling and trafficking^25^. Furthermore, the skeletal muscle-specific ablation of gamma-actin in mice progressively leads to a CNM-like disease^26^, suggesting that defects in actin dynamics may associate with myopathic phenotypes.

Here, we show that the GTP-ase activity of dynamin-2 is necessary to induce *de novo* actin polymerization and stimulus-elicited insertion of GLUT4 into the plasma membrane of muscle cells. Expression of CNM-associated dynamin-2 mutants suppresses the formation of new actin and inhibits the stimulus-dependent plasma membrane-insertion of GLUT4 in these cells. Consistently, myofibers isolated from heterozygous knock-in (KI) mice harboring the mutation p.R465W, an animal model of CNM^11^, exhibit reduced actin polymerization, altered actin organization and impaired insulin-induced insertion of GLUT4 into the sarcolemma. Further, we observed an abnormal perinuclear accumulation of GLUT4 in biopsies from CNM-patients harboring dynamin-2 middle domain mutations suggestive of trafficking defects.

These findings demonstrate the key role played by dynamin-2 in the regulation of the actin cytoskeleton in skeletal muscles and provide a better understanding of the pathomechanisms of dynamin-2-related CNM, through the negative impact of the CNM-associated dynamin-2 mutations on actin dynamics and actin-dependent trafficking in muscle cells.

## Results

### Dynamin GTP-ase activity is required for actin polymerization in RCMH myoblasts

**Figure 1:**
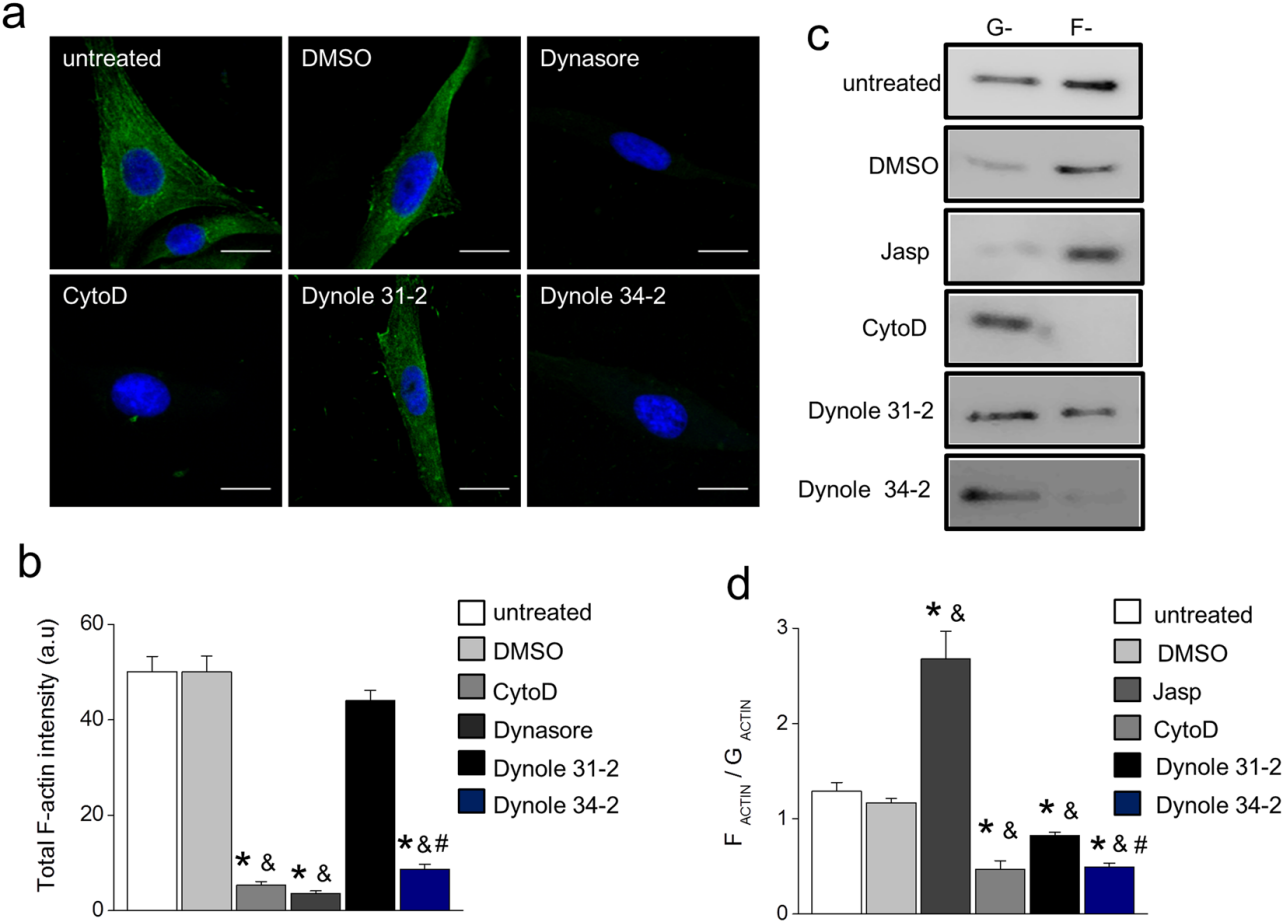
Dynamin GTP-ase activity regulates actin organization in RCMH myoblasts. (**a**,**b**) Cultured RCMH myoblasts were permeabilized during 6 min with 20 µM digitonin in the presence of G-actin-AF488, 2 mM ATP/Mg^2+^ and 10 µM free Ca^2+^, fixed and visualized by confocal microscopy. The pharmacological treatments applied during permeabilization were: 20 µM dynole 34-2, 20 µM dynole 31-2, 100 µM dynasore, 4 µM CytoD or the vehicle DMSO. (**a**) Examples of the formation of actin filaments in RCMH cells under different experimental conditions. Scale bar = 10 µm. (**b**) Quantification of total fluorescence intensity of new actin filaments. Note that dynamin GTP-ase inhibitors significantly reduce actin filament formation. Data are expressed as mean actin intensity ± SEM. Statistical comparisons were performed utilizing a two-tail t-test Welch corrected for parametric data. The symbols *, & and # denote significance compared to untreated, DMSO-treated and dynole 31-2-treated cells, respectively. N is between 15 and 28 cells, from at least three different cultures per condition. (**c**,**d**) Plated myoblasts were incubated during 15 min at 37 °C with 2 µM of the F-actin stabilizer jasplakinolide (Jasp), 4 µM CytoD, 20 µM dynole 34-2, 20 µM dynole 31-2 or the vehicle DMSO, then lysed with an actin-stabilizing buffer and ultracentrifuged to separate G- and F-actin. Samples were electrophored in 12% SDS-PAGE and developed by western blot with an anti-actin antibody; although not necessarily at the same time, all gels were run under the same experimental conditions. (**c**) Representative blots for G- and F-actin under different treatments. These are cropped images; full-length blots are shown in the Supplementary Fig. S1. (**d**) Quantification of band densities. Note that the F/G actin ratio increases in the presence of Jasp, while decreases in the presence of CytoD, as compared to DMSO-treated or untreated cells. Dynole 34-2 significantly reduced F/G ratio compared to its inactive analogue dynole 31-2. Data are expressed as means ± SEM from at least three different cultures per condition. Statistical comparisons were performed utilizing a two-tail Mann-Whitney non-parametric test. The symbols *, & and # denote significance compared to untreated, DMSO-treated and dynole 31-2-treated cells, respectively.

In order to evaluate the role of dynamin-2 in actin dynamics in muscle cells, we first assessed the *in vitro* formation of new actin in RCMH myoblasts, a cell line derived from a skeletal muscle biopsy of a healthy patient^27^. During the assay, cultured myoblasts were permeabilized with digitonin in the presence of a fluorescently-tagged monomeric actin (G-actin-AF488) and the total actin intensity of new formed filaments was measured as an estimate of *de novo* actin polymerization. To analyze the contribution of dynamin-2 to this process, two different pharmacological inhibitors of dynamin GTP-ase activity were used during permeabilization: dynasore and dynole 34-2. As shown in Fig. 1a and b, untreated RCMH myoblasts exhibited a total actin signal of 50.1 ± 3.2 arbitrary unit (AU, N = 26 cells). Statistically similar values were observed in myoblasts permeabilized in the presence of the vehicle DMSO (50.0 ± 3.4 AU, N = 23 cells) or 20 µM of the inactive agent dynole 31-2 (44.0 ± 2.2 AU, N = 23 cells). Conversely, the inhibition of dynamin GTP-ase activity with 100 µM of dynasore (3.6 ± 0.5 AU, N = 15 cells) or 20 µM of dynole 34-2 (8.6 ± 1.1 AU, N = 22 cells) significantly supressed the formation of new actin filaments compared to untreated cells (p < 0.0001) or cells treated with DMSO (p < 0.0001), suggesting a role of dynamin GTP-ase activity during actin polymerization in muscle cells. As expected, the disruption of actin with 4 µM of cytoclasin D (CytoD) significantly reduced the formation of actin filaments (5.3 ± 0.8 AU, N = 28 cells) compared to untreated (p < 0.0001) or DMSO-treated (p < 0.0001) cells (Fig. 1a,b).

To further evaluate the importance of dynamin GTP-ase activity on actin dynamics in myoblasts, we quantified the relative amounts of filamentous (F-) and monomeric (G-) actin in RCMH extracts lysed with an F-actin stabilizing buffer and previously treated with dynole 34-2 or dynole 31-2. We used the F-actin stabilizing drug jasplakinolide (Jasp) or CytoD as stabilization and depolymerization controls, respectively. Figure 1c shows representative cropped blots of all the pharmacological conditions analyzed. The respective full-length gels are shown in Supplementary Fig. S1. As expected, Jasp treatment significantly increased the F/G ratio in RCMH lysates (2.7 ± 0.3, N = 4 cultures) compared to untreated (1.3 ± 0.1, p < 0.01, N = 6 cultures) or DMSO-treated lysates (1.2 ± 0.04, p < 0.05, N = 4 cultures). By contrast, the F/G ratio was reduced in cells treated with CytoD (0.5 ± 0.1, N = 4 cultures) compared to untreated (p < 0.01) or DMSO-treated (p < 0.05) lysates (Fig. 1c,d), thus confirming the sensitivity of the assay. The treatment with dynole 34-2 modified the balance between F- and G- actin by diminishing the F/G ratio (0.5 ± 0.04, N = 8 cultures) compared to untreated (p < 0.001), DMSO-treated (p < 0.005) or to dynole 31-2-treated (p < 0.05, N = 6 cultures) lysates (Fig. 1c,d). It is noteworthy that, although the inactive analogue dynole 31-2 also decreased the F/G ratio (0.8 ± 0.04) compared to untreated (p < 0.005) or DMSO-treated lysates (p < 0.01) it is a necessary control for the treatment with dynole 34-2.

Taken together these results support the participation of dynamin GTP-ase activity in actin polymerization and organization in skeletal muscle cells.

### Dynamin’s GTP-ase activity is involved in GLUT4 trafficking in RCMH myoblasts

Considering the importance of actin remodeling in the intracellular trafficking^18, 19^, our next step was to evaluate whether actin-dependent trafficking is a process affected in muscle cells when dynamin function is compromised. To address this point, we focused on the mobilization of the glucose transporter GLUT4, a protein that continously cycles between the plasma membrane and intracellular compartments in response to insulin^28, 29^ and Ca^2+^-activated signalling pathways induced by contraction^30^.

**Figure 2:**
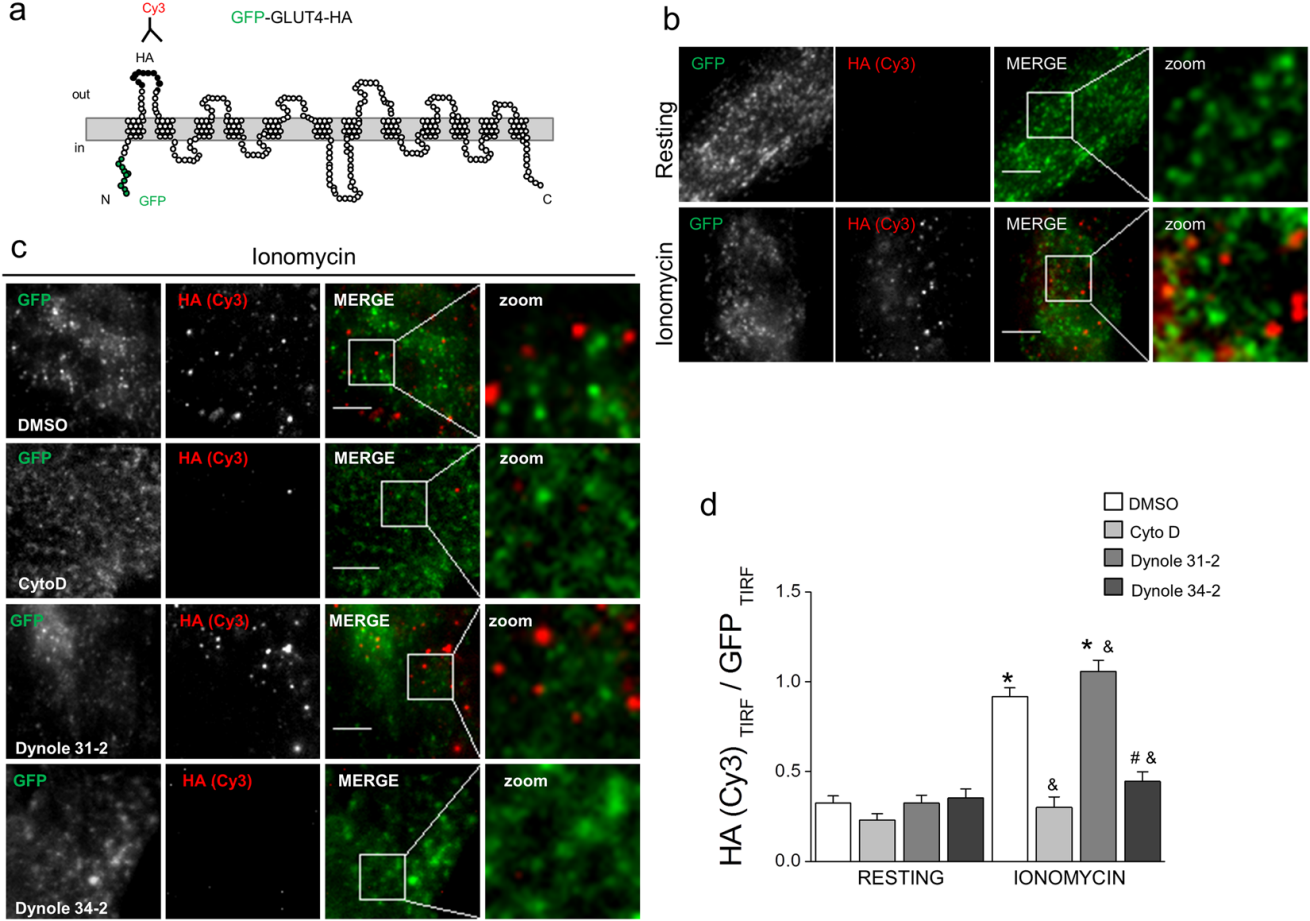
Pharmacological inhibition of dynamin GTP-activity reduces GLUT4 insertion in the plasma membrane of RCMH myoblasts. Cultured RCMH myoblasts were transfected with the GFP-GLUT4-HA construct and 48 ± 2 h later were stimulated for 5 min with 20 µM ionomycin in the presence of 20 µM dynole 31-2, 20 µM dynole 34-2, 4 µM CytoD or the vehicle DMSO at 37 °C, fixed, immunolabeled with an anti-HA antibody without permeabilization, incubated with a Cy3-conjugated antibody and visualized by TIRF microscopy. The plasma membrane insertion of GLUT4 was quantified as a ratio between Cy3 (red) and GFP (green) signals in the evanescent field. (**a**) Schematic representation of the GFP-GLUT4-HA construct; GFP is located in the N-terminal intracellular loop and the exofacial hemaglutinin (HA)-tag in the first extracellular loop. (**b**) Examples of TIRF images of non-treated cells under resting (upper panels) or stimulated (bottom panels) conditions (250 × 250 pixels images are shown). Note that ionomycin induces GLUT4 translocation, as the HA (Cy3) signal in the evanescent field becomes detectable (red spots). (**d**) 250 × 250 pixels representative images of RCMH cells stimulated in the presence of DMSO, CytoD, dynole 31-2 or dynole 34-2. Cell periphery is drawn in white. (**e**) HA (Cy3)/GFP ratio in the evanescent field is plotted for each experimental condition. Note that, as CytoD does, dynole 34-2 significantly inhibits the stimulus-dependent plasma membrane insertion of GLUT4. Data are means ± SEM. Statistical comparisons were performed utilizing a two-tail t-test Welch corrected for parametric data. The symbol * denote significance compared to the respective resting condition; & and # symbols denote significance compared to ionomycin-stimulated cells treated with DMSO and Dynole 31-2, respectively. N is between 20 and 29 cells, from at least three different cultures per experimental condition. Scale bar = 10 µm.

RCMH myoblasts were transfected with GFP-GLUT4-HA, a GLUT4 construct that express GFP and an exofacial hemaglutinin (HA) epitope which allowed us to visualize GLUT4 correctly inserted into the plasma membrane. The diagram in Fig. 2a represents the topology of GFP-GLUT4-HA in the plasma membrane. In order to by-pass the poor expression of insulin receptors in myoblast cultures^31^, we induced GLUT4 translocation by stimulating cells with the Ca^2+^ ionophore ionomycin. As shown in Fig. 2b, ionomycin stimulation notoriously promoted the plasma membrane insertion of GLUT4 in RCMH cells, as evidenced by an increase of the HA (Cy3) signal in the evanescent field using TIRF microscopy (Fig. 2b, bottom panels) compared to the basal state (Fig. 2b, top panels). Incubation with dynole 31-2 or DMSO did not interfere with the ionomycin-induced GLUT4 translocation (Fig. 2c,d), as we observed a significant increase in the HA (Cy3) signal in the evanescent field in stimulated DMSO-treated (0.9 ± 0.05 AU, p < 0.0001, N = 29 cells) and dynole 31-2-treated cells (1.1 ± 0.06 AU, p < 0.0001, N = 21 cells), compared to their respective resting conditions (0. 3 ± 0.04 AU, N = 21 cells for resting DMSO-treated cells and 0.3 ± 0.04 AU, N = 21 cells for resting Dynole-31-2-treated cells). Conversely, actin disruption with CytoD significantly inhibited the ionomycin-elicited plasma membrane insertion of GLUT4 (0.3 ± 0.06 AU, N = 21 cells), compared to that observed in DMSO-treated stimulated cells (p < 0.0001) (Fig. 2c,d). This latter is in agreement with previous reports where CytoD blocked the insulin-mediated GLUT4 translocation in L6-myocytes^32^. GTP-ase inhibition of dynamin-2 with dynole 34-2 also reduced the insertion of GLUT4 into the plasma membrane (0.4 ± 0.05 AU, N = 20 cells) compared to Dynole 31-2-treated (p < 0.0001) or DMSO-treated (p < 0.0001) stimulated cells (Fig. 2c,d), thus supporting the participation of dynamin GTP-ase activity in the stimulus-dependent GLUT4 translocation in muscle cells.

These data strongly suggest that dynamin GTP-ase activity is required for actin-dependent GLUT4 trafficking in muscle cells.

#### De novo actin polymerization is disrupted in RCMH myoblasts expressing CNM-linked dynamin-2 mutants

**Figure 3:**
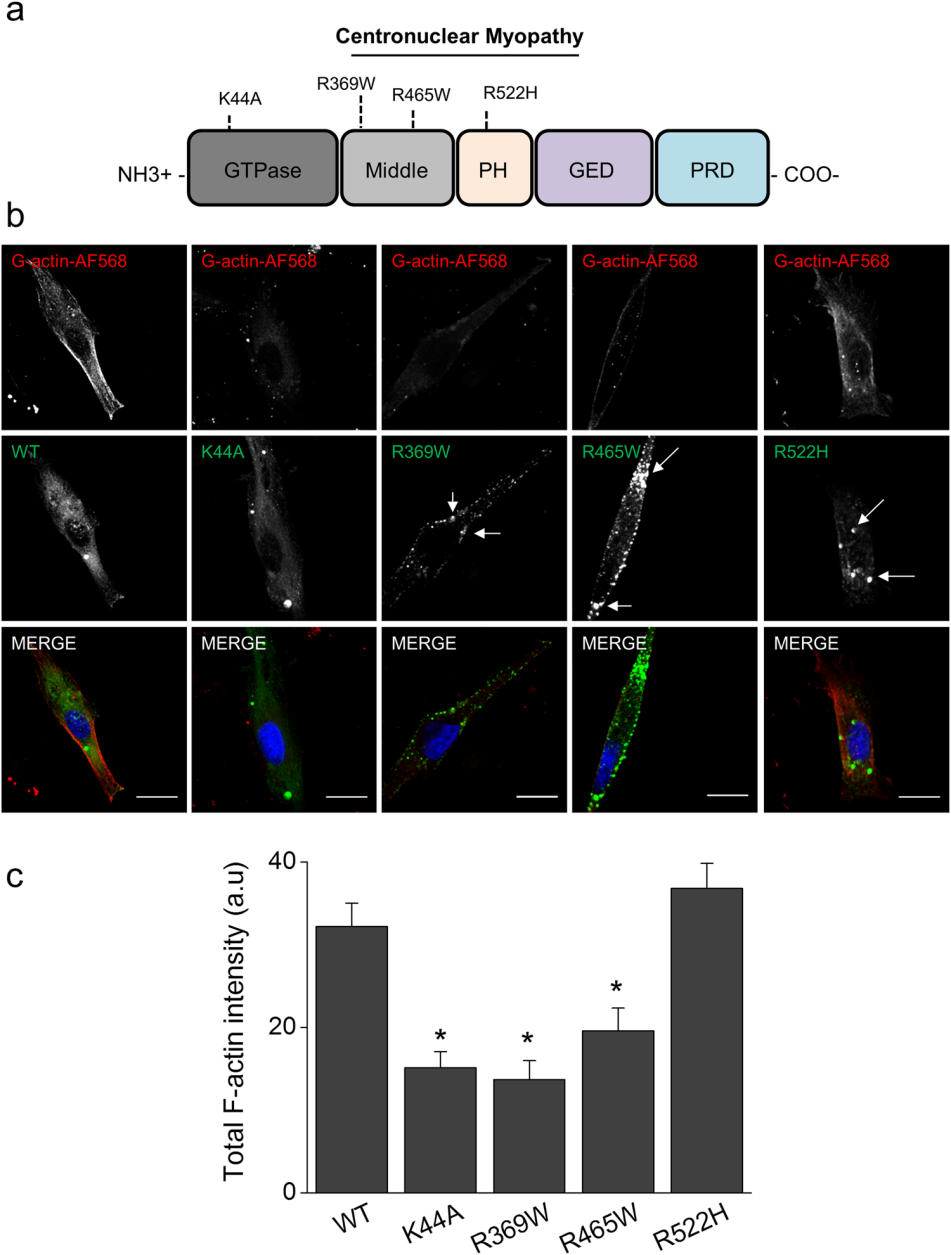
CNM-associated mutations in dynamin-2 reduce *de novo* actin filament formation in RCMH myoblasts. RCMH myoblasts were transfected by lipofection with EGFP-fused constructs expressing dynamin-2 WT, the GTP-ase defective mutant K44A, the middle domain mutants R369W or R465W or the PH domain mutant R522H. 48 h later, transfected cells were permeabilized with digitonin in the presence of G-actin-AF568, fixed and visualized by confocal microscopy. (**a**) The diagram indicates the location of the currently studied mutations. (**b**) Representative confocal images of the newly formed actin filaments (top panels) in RCMH myoblasts expressing Dyn2WT, K44A, R369W, R465W or R522H (middle panels). Note that CNM-mutants form dynamin-2 aggregates in the cytosol of RCMH cells (white arrows). Scale bar = 10 µm. (**c**) Quantification of the total intensity fluorescence of the recently formed actin. Note that, compared with WT, the expression of the mutants K44A, R369W and R465W, but not of R522H, significantly inhibits the new formation of actin filaments. Data are expressed as mean actin fluorescence intensity ± SEM. Statistical comparisons were performed utilizing a two-tail t-test Welch corrected for parametric data; *p < 0.0001 compared to Dyn2WT-transfected cells. N is between 24 and 34 cells, from at least three different cultures per experimental condition.

In order to determine how CNM-causing mutations impact on the role of dynamin in actin polymerization and actin-mediated trafficking in muscle cells, we transfected RCMH myoblasts with EGFP-fused constructs of wild type dynamin-2 (WT) or the dynamin-2 CNM-linked mutants R369W, R465W or R522H. In parallel, we used the construct GFP-K44A, a dynamin-2 mutant defective in GTP-binding and hydrolysis^33^. A schematic representation of the location of the currently studied mutations is shown in Fig. 3a; p.K44A localizes in the GTP-ase domain of dynamin-2, p.R369W and p.R465W localize in its middle domain and p.R522H is located in the amino-terminal region of the PH domain.

WT exhibited a homogeneous distribution within the cytosol of RCMH cells (N = 34 cells) and a similar pattern was observed in cells expressing K44A (N = 30 cells, Fig. 3b). However, R369W (N = 24 cells), R465W (N = 25 cells) and R522H (N = 25 cells) formed cytosolic inclusions (Fig. 3b), according to the increased aggregation capability of these mutants^34–36^.

To analyze the impact of these mutants on actin polymerization, we permeabilized transfected RCMH myoblasts in the presence of G-actin-AF568. As shown in Fig. 3b (top panels), formation of new actin filaments was observed in myoblasts expressing WT with a mean actin-fluorescence signal of 32.2 ± 2.8 AU. Conversely, in myoblasts transfected with the mutant K44A, we observed a significantly reduced formation of actin filaments (15.1 ± 1.9 AU) compared to WT-transfeted cells (p < 0.0001; Fig. 3b,c). This result is in agreement with our prior observations in the presence of dynasore or dynole 34-2 (Fig. 1a). RCMH cells transfected with R369W and R465W mutants also exhibited a diminished capability to form new actin filaments (13.7 ± 2.3 AU for R369W-transfected cells and 19. 6 ± 2.8 AU for R465W-transfected cells) as compared to cells expressing WT (p < 0.005; Fig. 3b,c). On the other hand, the formation of new actin filaments was not signficantly affected in cells expressing the R522H mutant (36.8 ± 3.1) compared to WT-transfected cells (Fig. 3b,c). The latter suggests that not all CNM-linked mutants have the same impact on dynamin-2-dependent actin dynamics.

#### CNM-causing dynamin-2 mutations inhibit stimulus-dependent translocation of endogenous GLUT4 in RCMH myoblasts

In order to analyze the impact of CNM-linked dynamin-2 mutations in actin-dependent trafficking, we assessed the stimulus-induced translocation of endogenous GLUT4 in RCMH myoblasts transfected with R369W, R465W or R522H. To determine the effect of the disruption of dynamin-2 GTP-ase activity in this process, we transfected cells with the K44A mutant. Transfected cells were stimulated during 5 min with 20 µM ionomycin to induce GLUT4 translocation towards plasma membrane. After stimulation, cells were fixed, immunolabeled with a specific GLUT4 antibody and visualized by TIRF microscopy. GLUT4 insertion into the plasma membrane was measured by quantifying the total fluorescence intensity of the GLUT4 signal in the evanescent field.

**Figure 4:**
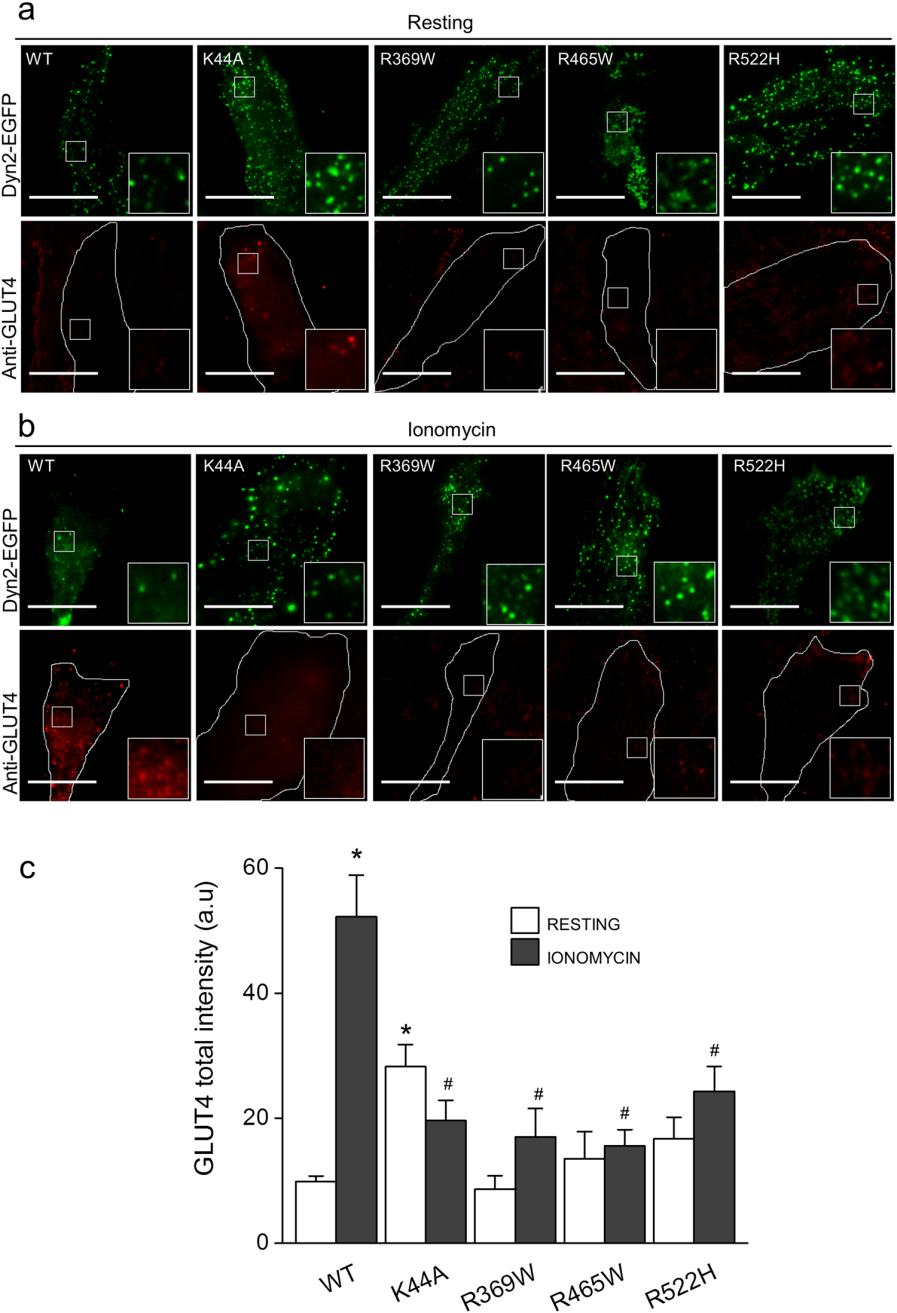
Dynamin-2-CNM-causing mutations reduce stimulus-induced translocation of endogenous GLUT4 in RCMH myoblasts. RCMH myoblasts were transfected with the dynamin-2 EGFP-fused constructs WT, K44A, R369W, R465W or R522H. 48 h later, transfected cells were stimulated with 20 μM ionomycin during 5 min, fixed, immunolabeled with a monoclonal antibody directed against GLUT4 and visualized by TIRF microscopy. Plasma membrane insertion of endogenous GLUT4 was quantified as the total intensity fluorescence of GLUT4 in the evanescent field. (**a**,**b**) Examples of TIRF images (250 × 250 pixels) of RCMH myoblasts at the resting (**a**) and ionomycin-stimulated condition (**b**). Cell periphery is drawn in white in GLUT4 panels. (**c**) Quantification of GLUT4 total intensity fluorescence in the evanescent field. Note that cells expressing K44A mutant exhibit significantly higher levels of GLUT4 at the resting condition compared to resting-cells expressing Dyn2WT. Ionomycin increased GLUT4 signal in Dyn2WT-transfected cells but was not enough to increase GLUT4 signal in myoblasts expressing all the mutated versions of dynamin-2. Data are expressed as the mean GLUT4 total intensity fluorescence ± SEM. Statistical comparisons were performed utilizing a two-tail t-test Welch corrected for parametric data. The symbols * and # denote significance compared to Dyn2WT-transfected resting cells and Dyn2WT-transfected stimulated cells respectively. N is between 18 and 35 cells, from at least three different cultures per experimental condition.

As shown in Fig. 4a, the endogenous GLUT4 signal in the evanescent field was weak in resting cells expressing the WT construct (N = 18 cells), with a mean signal of 9.9 ± 3.3 AU (Fig. 4c), however, a significantly higher GLUT4 signal (28.2 ± 3.5 AU, p < 0.001, N = 20 cells) was observed in resting cells expressing the K44A mutant (Fig. 4a,c). As dynamin-2 activity is an important regulator of GLUT4 endocytosis in muscle cells^37–39^, such augmented GLUT4 signal in plasma membrane at the basal state could be consequence of overall deficiencies in endocytosis. GLUT4 signal in the evanescent field was not significantly different from WT-transfected cells in myoblasts expressing R369W (8.6 ± 2. 1 AU, N = 19 cells), R465W (13.5 ± 4. 3 AU, N = 19 cells) or R522H mutants (16.7 ± 3. 4 AU, N = 24 cells) at the resting condition (Fig. 4a,c).

Compared to the basal state, stimulation with ionomycin significantly increased the GLUT4 signal in the evanescent field in cells expressing WT dynamin-2 (52.2 ± 6.6 AU, p < 0.0001, N = 35 cells), as an indication of GLUT4 insertion into the plasma membrane (Fig. 4c). On the contrary, in RCMH myoblasts expressing the K44A mutant, ionomycin proved insufficient to promote plasma membrane insertion of GLUT4, as no significant changes were observed in the GLUT4 signal in stimulated cells (19.6 ± 3.2 AU, N = 24 cells) compared to the respective resting condition (Fig. 4c). This result is in agreement with the reduced insertion of GLUT4 observed in cells treated with dynole 34-2 (Fig. 2). RCMH myoblasts expressing R369W (N = 22 cells), R465W (N = 29 cells), or R522H mutants (N = 26 cells), also exhibited a diminished plasma membrane insertion of GLUT4 in response to ionomycin-stimulation (17.03 ± 4.5 AU for R369W; 15.6 ± 2.6 AU for R465W and 24.3 ± 3.9 AU for R522, respectively; p < 0.001), compared to WT-transfected cells (Fig. 4c). In order to discard that the effect of the CNM mutants on GLUT4 translocation is a consequence of a differential expression of the dynamin-2 constructs, we normalized the GLUT4 signal in TIRF by the respective EGFP total intensity in epifluorescence. Using this strategy, we observed a similar decrease in the plasma membrane insertion of GLUT4 in cells transfected with the CNM mutants, compared to WT-transfected cells (Supplementary Fig. S6).

Taken together, these results support the idea that actin-mediated intracellular trafficking is deregulated in muscle cells expressing CNM-causing dynamin-2 mutations.

#### Actin organization is modified in muscle of HTZ KI-R465W mice, an animal model of CNM

To test the possibility that actin-dependent trafficking is affected in CNM, we used a KI mouse line expressing the p.R465W mutation, a mammalian model of CNM^11^. Heterozygous KI mice (HTZ) recapitulate signs of myopathy, including impairment of the muscle fiber contractile properties and atrophy^11^. In this animal model, muscle atrophy started at 2 months of age and then slowly worsened^11^. Therefore, and in order to understand early pathological mechanisms, we analyzed actin alterations in animals of 2–3 months.

**Figure 5:**
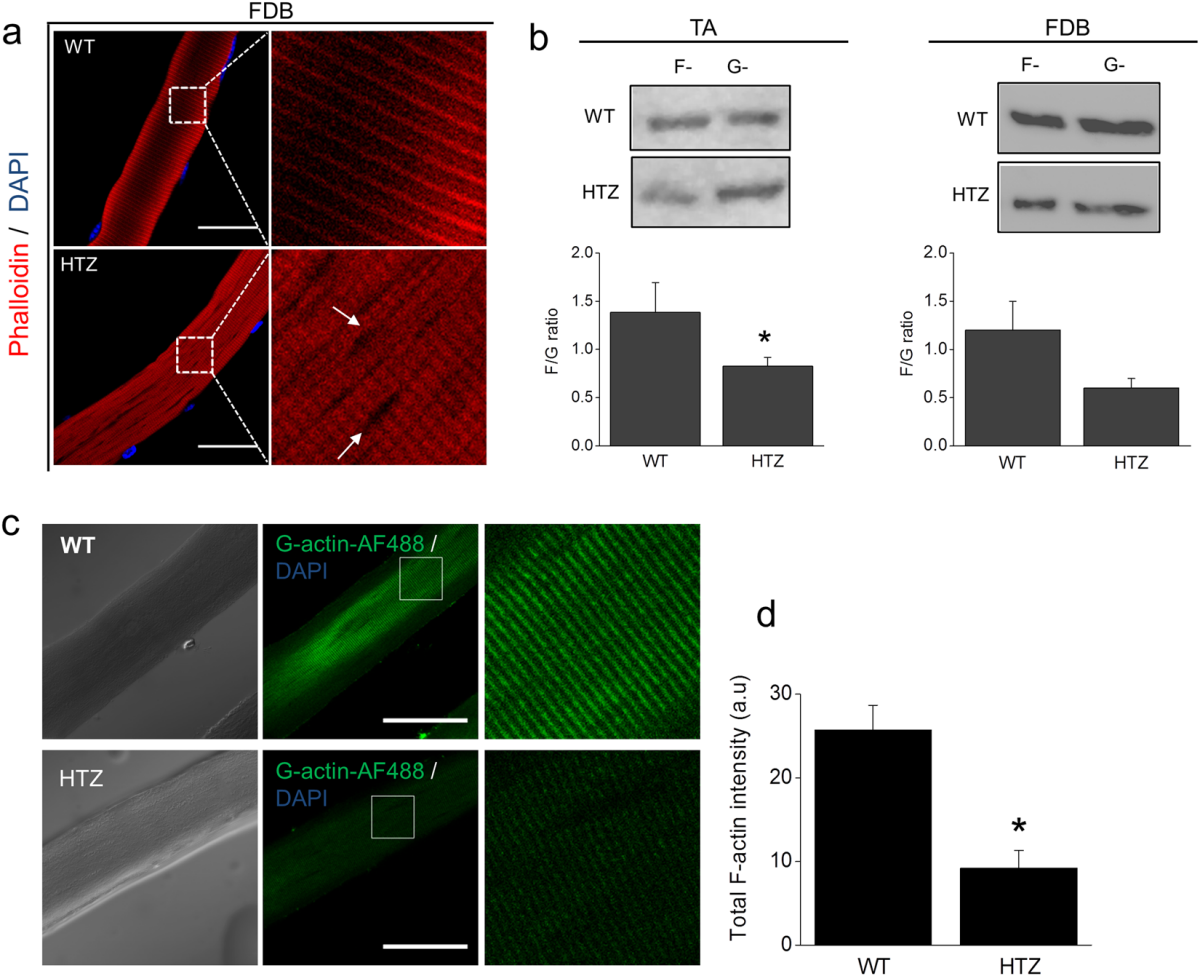
Actin organization and polymerization is altered in muscles of HTZ mice harboring the mutation R465W. (**a**) FDB muscles dissected from 2 month-old HTZ and WT mice were digested with collagenase. Fibers were isolated, fixed in PFA, permeabilized and stained with phalloidin-Rhodamine-B to visualize the actin network. Myonuclei were stained with DAPI. Representative confocal images of WT (top panels) and HTZ (bottom panels) fibers are shown. White square enclose the enlarged areas. Notice that phalloidin-staining looks altered in HTZ myofibers, exhibiting non-stained areas that did not localize with DAPI-stained nuclei (white arrows). Scale bar = 20 µm; n is between 48 and 50 fibers from eight different animals per genotype. (**b**) To evaluate the relative amounts of F- and G-actin, freshly dissected *tibialis anterior* (TA, left) and FDB muscles (right) were lysed with a commercial F/G actin *in vivo* assay, electrophorated by 12%-SDS-PAGE and revealed by western blot using a polyclonal actin antibody. Above are shown representative blots for F- and G-actin per muscle and genotype; these are cropped images and the original full-length blot is shown in Supplementary Fig. S2. Below are plotted the F/G actin ratios per genotype. Note that F/G tend to be lower in HTZ muscles compared to WT muscles in both TA and FDB, although this difference is only significant in TA muscles. Data are expressed as the mean F/G ratio ± SEM for muscles from at least three animals per genotype. Statistical comparisons were performed utilizing a two-tail Mann-Whitney non-parametric test. * denote significance with respect to WT-muscles. (**c**,**d**) To evaluate actin polymerization, isolated FDB myofibers were stimulated with 0.1 µM insulin during 10 min at 37 °C, permeabilized with digitonin in the presence of G-actin-AF488, fixed and visualized by confocal microscopy. (**c**) Representative images of newly formed actin filaments in WT and HTZ fibers. Left panels show the respective DIC images. Scale bar = 20 µm. (**d**) Quantification of total G-actin-AF488 fluorescence intensity. Note that HTZ fibers exhibit a decreased capability to form new actin filaments compared to WT fibers. Data are expressed as mean actin signal ± SEM. Statistical comparisons were performed utilizing a two-tail Mann-Whitney non-parametric test. The symbol * denote significance with respect to WT-fibers. N is between 24 and 25 fibers from five different animals per genotype.

We first evaluated the impact of the p.R465W mutation on the overall actin organization pattern in mature fibers isolated from *flexor*-*digitorium brevis* (FDB) muscles of WT and HTZ mice. The isolated fibers were stained with the toxin phalloidin. Figure 5a shows representative images of WT and HTZ fibers. In WT animals 84% of the analyzed fibers (n = 50 fibers) showed a regular striated pattern when stained with phalloidin (Fig. 5a, top panels), whilst in HTZ animals only 33% of the fibers analyzed (n = 48) exhibited a regular actin organization. Conversely, 66% of the HTZ myofibers exhibited irregular phalloidin labeling with incomplete staining areas, which did not colocalize with DAPI-stained nuclei (Fig. 5a, white arrows), suggesting the presence of modifications in actin organization and structural alterations in HTZ myofibers.

To determine whether changes in the actin organization pattern involved modifications in the relative amounts of F- and G-actin, we quantified the F/G actin ratio in lysates from freshly dissected *tibialis anterior* (TA) *and FDB* muscles of WT and HTZ mice. Representative cropped blots are shown in Fig. 5b. Full length gels are shown in Supplementary Fig. S2. As observed, the F/G actin ratio was significantly decreased in TA extracts of HTZ mice (0.83 ± 0.09, N = 5 animals, p < 0.05), compared to WT animals (1.4 ± 0.3, N = 5 animals). In FDB muscles, we also observed a tendency to decrease the F/G ratio in HTZ (0.6 ± 0.2, N = 3 animals) compared to WT mice (1.2 ± 0.3, n = 3 animals), although by the observed variability this difference was not significant.

To analyze whether the formation of new actin filaments is affected in HTZ muscles, we performed the *de novo* actin polymerization assay described above (Figs 1 and 3). In this case, isolated FDB fibers were stimulated with 0.1 µM insulin prior to perform the assay, as reportedly this hormone induces actin remodeling in muscle cells^24^. As shown in Fig. 5c, insulin induced the formation of new actin filaments in WT FDB fibers (Fig. 5c, top panels), which exhibited a mean total actin intensity of 25.7 ± 2.9 AU (N = 25 fibers). However, it was significantly less efficient in to promote actin polymerization in HTZ fibers (Fig. 5c,d), reaching a mean actin intensity value of 9.2 ± 2.1 AU (p < 0.0001, N = 24 fibers). Mean total G-actin-AF488 fluorescence intensities are ploted in Fig. 5d. These data suggest that a limited actin remodeling could underlie deregulated mechanisms in CNM.

#### Insulin-induced GLUT4 translocation is disrupted in muscle fibers isolated from HTZ KI-R465W mice

In order to investigate whether GLUT4 trafficking is affected in HTZ animals, we analyzed the distribution of endogenous GLUT4 in FDB myofibers isolated from WT and HTZ mice.

**Figure 6:**
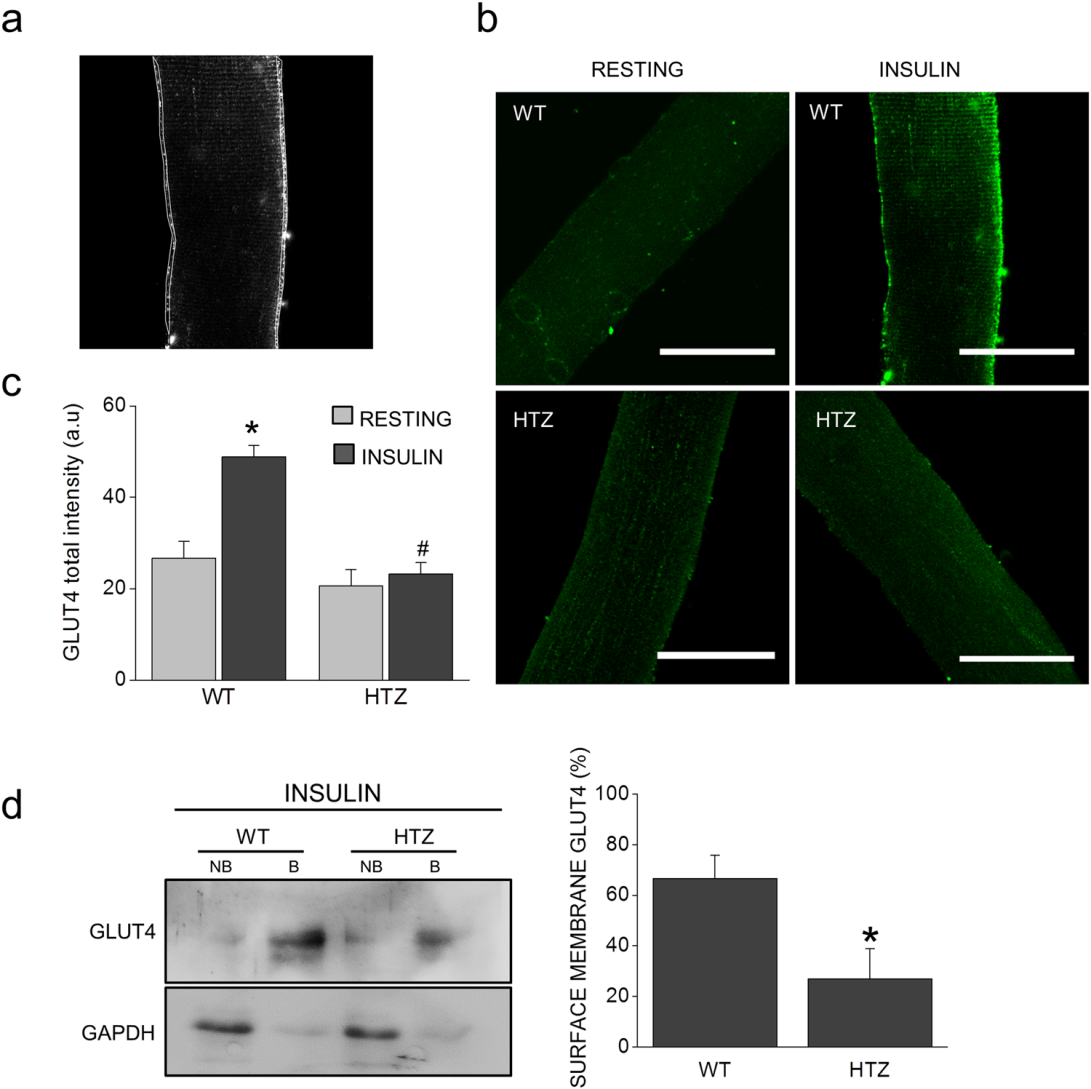
Insulin-induced translocation of GLUT4 is disrupted in muscle fibers isolated from HTZ mice. (**a–c**) Freshly dissected FDB muscles from 2 month-old WT and HTZ mice were digested with collagenase. Isolated fibers were stimulated during 15 min with 0.1 µM insulin to induce GLUT4 translocation, fixed and immunolabeled with a polyclonal-GLUT4 antibody. Translocation of GLUT4 was estimated by measuring the total intensity fluorescence of GLUT4 in ROIs at the sarcolemma. (**a**) Examples of the ROIs used are drawn in white. GLUT4 was measured on both edges of the confocal image and then averaged. (**b**) Examples images of GLUT4 signal in WT and HTZ fibers at the resting (left panels) and insulin-stimulated condition (right panels). Scale bar = 20 µm. (**c**) The graph show the averaged GLUT4 signal in sarcolemma. Note that insulin-induced GLUT4 translocation is significantly reduced in HTZ myofibers compared to WT myofibers. Data are expressed as mean GLUT4 fluorescence signal ± SEM. Statistical comparisons were performed utilizing a two-tail t-test Welch corrected for parametric data. The symbols * and # denote significance with respect to WT-resting and WT-insulin-stimulated fibers, respectively. N is between 34 and 66 fibers from at least 10 different animals per genotype. (**d**) FDB muscles were dissected from WT and HTZ mice, stabilized in Tyrode solution, stimulated for 30 min with 0.1 µM insulin and then exposed to 1 mg/ml of biotin at 4 °C during 60 min. After quenching with 100 mM glycine, muscles were frozen and pulverized in liquid nitrogen, lysed and centrifuged at 14.000 g for 10 min. Supernatants were mixed with streptavidin-agarose beads overnight at 4 °C and then centrifuged at 14.000 g for 3 min. Biotinylated and non-biotinylated fractions were used to evaluate GLUT4 expression by western blot. GAPDH was used as a control that only surface proteins were labeled in biotinylated fractions. On the left are shown representative blots per each condition, on the right are plotted the percentages of GLUT4 in biotinylated fractions. Data are expressed as mean GLUT4% ± SEM. Statistical comparisons were performed utilizing a two-tail t-test Welch corrected for parametric data. The symbol * denote significance with respect to WT-muscles. N is five different animals per genotype.

Briefly, freshly isolated fibers were stimulated during 15 min with 0.1 µM insulin, then fixed and immunostained for GLUT4 localization. The total fluorescence intensity of ROIs along the sarcolemma was measured in order to estimate the amount of GLUT4 inserted into the surface membrane. Figure 6a shows an example of the ROIs used for quantification of the GLUT4 signal in sarcolemma. The total fluorescence intensity of both sarcolemma edges were averaged and normalized to the ROI area. As observed in Fig. 6b, at the resting state, endogenous GLUT4 localized diffusely throughout the entire fiber in both WT and HTZ fibers (left panels). Fluorescence intensity of GLUT4 was not significantly different at the sarcolemma of resting WT (N = 34) and HTZ-fibers (N = 42), reaching values of 26.7 ± 4.1 AU and 21.1 ± 4.1 AU, respectively. After 15 min of insulin stimulation, WT fibers exhibited a marked GLUT4 staining along the sarcolemma (Fig. 6b, right panels) reaching values significantly higher than those of the resting condition (49.7 ± 3.5 AU, p < 0.0001, N = 66 fibers). However, upon insulin stimulation of HTZ fibers, no significant change in GLUT4 intensity was observed (21.3 ± 3.3 AU, N = 49 fibers) as compared to the basal state (Fig. 6b,c). The latter suggests a deficient insulin-induced trafficking of GLUT4 vesicles in isolated HTZ muscle fibers. To confirm this hypothesis, we analyzed the expression of GLUT4 in biotinylated fractions of insulin-stimulated FDB muscles. Figure 6d shows that, after insulin stimulation, GLUT4 is enriched in biotinylated fractions (“B” lanes) of skeletal muscles from both WT and HTZ-KI mice. However, GLUT4 expression was significantly reduced in surface membranes of HTZ KI-stimulated muscles (26.9 ± 11.9%, p < 0.05, N = 5 animals) compared to WT-stimulated muscles (66.6 ± 9.3%, N = 5 animals), further suggesting an impaired insulin-induced translocation of GLUT4 in myofibers of this animal model.

#### GLUT4 is abnormally distributed in skeletal muscle of CNM-patients

**Figure 7:**
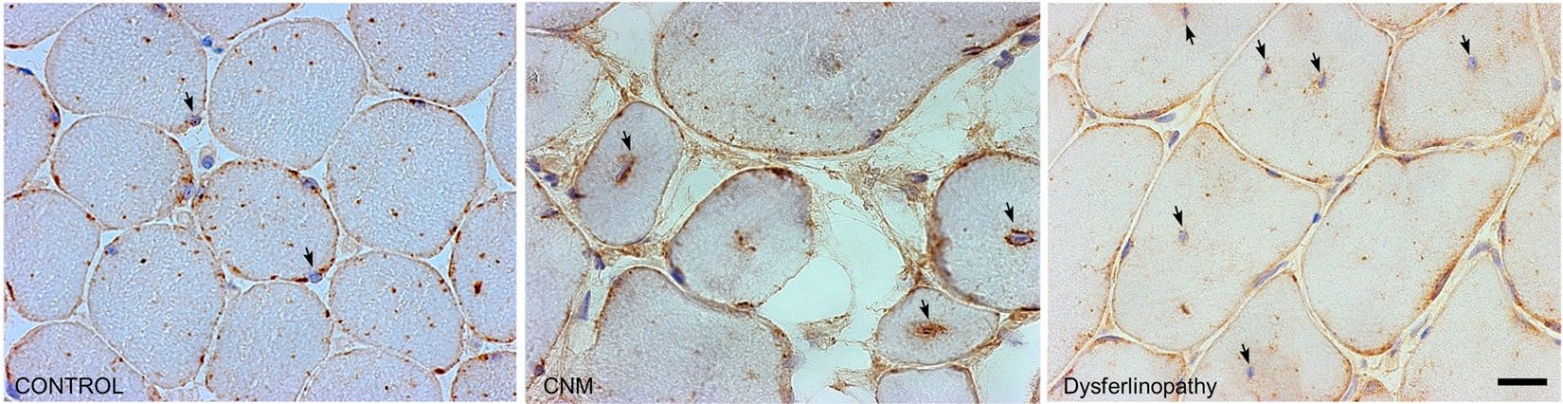
Abnormal perinuclear accumulation of GLUT4 in CNM skeletal muscles. Muscle biopsies from one non affected subject (control), one patient carrying the dynamin-2 mutation p.R465W (CNM) and one patient harboring a mutation in dysferlin (dysferlinopathy) were immunolabeled with a specific antibody against GLUT4 and visualized by field microscopy. In the control biopsy (left panel) GLUT4 staining is observed in sarcolemma and sarcoplasm without any particular distribution. A similar GLUT4 distribution is observed in the disferlinopathy samples (right panel); note that, even in fibers in which nuclei are centrally located (black arrows), there is not GLUT4 accumulation near nuclei in the disferlinopathy biopsy. In the CNM patient biopsy (middle panel) GLUT4 is at the sarcolemma, but strongly concentrates around nuclei in fibers with nuclear centralization (magnification x20).

Finally, we evaluated the subcellular localization of GLUT4 in skeletal muscle biopsies from CNM-patients carrying two middle domain mutations in dynamin-2: p.R465W and p.R369Q. We compared these with biopsies from a same-aged healthy subject (control) and from patients with muscular dystrophy caused by mutations in the dysferlin gene (dysferlinopathy) or in the dystrophin gene (dystrophinopathy). As shown in Fig. 7 (CNM panel) and in the Supplementary Fig. S5 (D–F panels), muscle biopsies from CNM patients exhibited centrally located nuclei and an abnormal perinuclear accumulation of GLUT4 (black arrows). This altered GLUT4 distribution was not observed in muscle biopsies from dysferlinopathy (Fig. 7; Fig. S5, panel B) or dystrophinopathy patients (Fig. S5, C panel) where the GLUT4 labeling mainly localizes in the periphery of the fibers, as observed in the control healthy subject (Fig. 7; Fig. S5, panel A). These findings are suggestive of the defects herein described are specifically associated to dynamin-2 mutations.

## Discussion

Dynamin-2 is a large GTP-ase involved in remodeling of membranes and actin cytoskeleton during different cellular processes^1–4, 40^. In spite of the ubiquitous expression of dynamin-2, CNM-linked mutations cause a skeletal muscle-specific disease, suggesting that muscle structure and function importantly depend on dynamin-2 activity. However, the pathogenic mechanism of dynamin-2-related CNM is still unclear.

Here, we have demonstrated that dynamin-2 is a key regulator of the actin dynamics and actin-dependent trafficking in muscle cells, and that these processes are susceptible to be impaired under conditions affecting dynamin-2 function. Regarding actin dynamics, our findings show that the pharmacological disruption of dynamin GTP-ase activity reduces the F/G actin ratio (Fig. 1c,d) and suppresses the formation of new actin filaments in myoblasts (Fig. 1a,b). Although the two dynamin inhibitors used here (dynasore and dynole 34-2) had effects similar to those produced by the dynamin-2 mutant K44A (Fig. 3), we cannot discard the possibility that these pharmacological agents have off-target effects on actin polymerization. In this regard, dynasore *per se* has been shown to influence F-actin stability^41^ and Rac activation^42^. Hence, it is possible that dynasore disrupts actin polymerization in RCMH myoblasts in a way that not only depends on dynamin inhibition. In spite of this, dynamin-2 GTP-ase activity appears to be critical to promote the formation of new actin filaments in muscle cells (see the effect of the GTP-ase defective mutant K44A in Fig. 3). This regulation may result from the well described association of dynamin-2 with F-actin binding proteins^6, 7, 43^, or alternatively from a direct interaction between dynamin and actin^5^. Regarding the latter mechanism, Sever and collaborators suggested the existence of a putative actin-binding motif in the middle domain of dynamin. This region would allow the direct association of dynamin to short actin filaments^5^. The proposed model suggests that unassembled dynamins bind to short actin filaments, oligomerize in rings that promote their GTP-ase activity and favor F-actin elongation^5^. These two levels of regulation need to be further investigated in muscle cells and tissue.

Concerning the effects of CNM-linked mutations on F-actin dynamics, we found that the middle-domain mutations p.R369W and p.R465W disrupt the ability of dynamin-2 to promote actin remodeling (Fig. 3). Interestingly, it was not observed in RCMH cells expressing the PH-domain mutation p.R522H. Why could actin dynamics be more susceptible to mutations located in the middle domain of dynamin-2? p.R369W and p.R465W localize near to the putative actin-binding motif, mapped between the residues 399 and 444^5^ (the orientation of these CNM mutations in the crystal structure of human dynamin-1 is shown in the Supplementary Fig. S3). Therefore, substitution of an arginine residue by tryptophan in either 369 or 465 position could influence actin-binding properties of dynamin-2 affecting actin polymerization. As actin binding promotes dynamin oligomerization and catalytic activity^44^, p.R369W and p.R465W mutations could additionally disrupt the actin-induced GTP-ase activity of dynamin-2. On the other hand, the p.R522H mutation localizes in the amino-terminal region of the PH domain (the orientation of the R522 residue in the crystal of human dynamin-1 is shown in Fig. S3). This region contains a hydrophobic variable-loop required for dynamin insertion into lipid membranes^45^ and for its self-assembly-induced activity^46^. Thus, it is likely that the R522H mutation impacts on dynamin-2 activity by interfering with its lipid-binding properties, and to a lesser extent on its actin-binding properties.

Besides the effect of the CNM-mutants on actin formation in RCMH cells (Fig. 3), we also observed a reduced polymerization and decreased F/G actin ratio in myofibers (Fig. 5b,c) and in primary culture myoblasts (Supplementary Fig. S4a) from HTZ mice harboring the mutation p.R465W. Moreover, myofibers from HTZ mice exhibit an irregular phalloidin-staining (Fig. 5a), which is suggestive of actin disorganization. Regarding this observation, we cannot discard that a phalloidin-altered pattern is due to structural disorganizations. In fact, both F-actin and dynamin-2 are required for focal adhesion assembly/disassembly, which in skeletal muscle fibers are concentrated at specialized adhesives structures critical to maintain the sarcomere integrity^47^. As the sarcomere stability is highly influenced by its association to costameres^48^, which are muscle-specific adhesion sites enriched in actin^49^ and dynamin-2^50^, it is possible that focal areas of disorganization in HTZ-mouse myofibers are a consequence of an altered costamere organization. The latter might be yet another mechanism that contributes to the pathogenesis of CNM caused by dynamin-2 mutations.

As actin dynamics regulates intracellular trafficking^18, 19^, dynamin-2 activity should influence trafficking pathways required to maintain an adequate muscle function^17^. To test this hypothesis, we studied the translocation of GLUT4, a process that requires a dynamic remodeling of the actin network^24, 32, 51^. GLUT4 follows a well-characterized actin-dependent trafficking pathway from sub-sarcolemmal vesicle pools that enable its rapid recruitment to the plasma membrane under stimulation conditions^24, 32, 51^. The subsequent retrieval of GLUT4 occurs via clathrin- and dynamin-2-dependent endocytosis^37–39^. Here we show that, in muscle cells, dynamin GTP-ase activity is not only necessary for GLUT4 endocytosis as previously described^37–39^, but it is also required for stimulus-induced insertion of GLUT4 into the plasma membrane. We found that RCMH myoblasts expressing the R369W, R465W or R522H mutants (Fig. 4), as well as primary culture myoblasts (Supplementary Fig. S4b) and myofibers (Fig. 5) from HTZ mice exhibit an impaired stimulus-dependent GLUT4 insertion into the plasma membrane. As distinct CNM-linked dynamin-2 mutations appear to disturb actin remodeling differently, it is probable that these mutations impair plasma membrane insertion of GLUT4 via different mechanisms. In this regard, the efficient expression of GLUT4 in plasma membrane also depends on the formation and recycling of intracellular pools of GLUT4-containing vesicles^52, 53^. The continuous cycling of GLUT4 involves anterograde and retrograde trafficking routes throughout endosomes and the trans-Golgi network (TGN)^52, 53^. In insulin-responsive tissues, GLUT4 accumulates in small storage vesicles (GSVs) as well as in larger storage sites including specializations of the TGN and recycling endosomes^54^. Upon insulin stimulation, translocation of GLUT4 to the cell surface is promoted by both the release of GSVs as well as by an accelerated inter-endosomal transit^54^. Since dynamin-2 participates in the emergence of new vesicles from endosomes^55^ and TGN^56, 57^, its disruption at this level might also influence the subcellular distribution of GLUT4. To address this issue, additional experiments are required to determine the impact of CNM-causing dynamin-2 mutations on the different stages of the GLUT4 recycling pathway.

The internalization rate of GLUT4 is another critical regulation-point for its plasma membrane localization^53^ and, speculatively, dynamin-2-dependent GLUT4 endocytosis could be also influenced by CNM-causing mutations. However, is still controversial whether all CNM-mutations impair endocytosis. In fact, defects in clathrin-mediated endocytosis were reported in cultured cells overexpressing some CNM-mutants^58, 59^, but not confirmed in other studies^60, 61^. From these data, it appears that results may depend on the dynamin-2 expression level, the cargo, or the cell type. So far, there is no evidence of the impact of these mutations on endocytosis in muscle cells, a possibility that is well worth studying in the future.

In addition to altered translocation, endocytosis and recycling, it is also possible that CNM-linked mutations may produce disorganization of the GLUT4-positive compartments in muscle cells, in turn affecting its expression in surface membranes. In this regard, we now show an abnormal peri-nuclear accumulation of the GLUT4 staining in biopsies from skeletal muscles of CNM-patients harboring the middle domain mutations p.R465W and p.R369Q (Fig. 7 and Supplementary Fig. S5). Although these observations are suggestive of intracellular trafficking defects, we cannot exclude the possibility that this pattern is a consequence of an intermyofibrillar reorganization of different GLUT4-containing structures. Indeed, one of the most characteristic features of the CNM-muscles is a halo of disorganized cytoplasmic organelles surrounding centrally located myonuclei^62, 63^.

Another plausible mechanism for the impairment in actin dynamics and GLUT4 is a decreased availability of the dynamin-2 CNM-mutants caused by their oligomerization at abnormal cellular sites^64^. Pioneer studies on physical and enzymatic properties of dynamin-2 showed that some middle- and PH-domain mutants exhibit a greater propensity to self-assemble forming larger oligomers that lead to increased basal GTPase activity compared to the WT protein^34, 35^. These larger oligomers localize at the plasma membrane as well as in the cytoplasm^36^. Abnormal cytosolic accumulation of dynamin-2 has been also observed in myofibers from CNM patients carrying a PH-domain mutation^65^ and in fibers isolated from HTZ mice^11^. We also observed large intracellular inclusions in myoblasts expressing R369W, R465W or R522H mutants (Fig. 3, white arrows), suggesting that aberrant oligomerization of these mutants could also contribute to the defects observed in actin dynamics (Fig. 3) and GLUT4 trafficking (Fig. 4) a point that certainly needs to be investigated.

Finally, the defects that we observed in actin dynamics and GLUT4 trafficking could constitute pathological mechanisms that potentially could explain some of the clinical features in dynamin-2-dependent CNM. Regarding cytoskeletal actin dynamics, it has been demonstrated that the specific ablation of gamma-actin causes a CNM-like phenotype in mice, characterized by progressive muscle weakness and necrosis^26^. Cytoskeletal actin alterations can modify trafficking and localization of a number of sarcolemmal proteins impacting on the skeletal muscle function. In fact, actin polymerization is required not only for GLUT4 transportation in muscle cells but also for clustering acetylcholine receptors at the neuromuscular junction^66^ and to recruit dysferlin to wounded areas of the sarcolemma^67^, supporting the idea that cytoskeletal actin disruption can lead to diverse muscle disorders by impairing intracellular trafficking.

Regarding GLUT4 trafficking, a deficient expression of GLUT4 in surface membranes could lead to defects in glucose uptake and metabolism, which in skeletal muscles, mainly depend on GLUT4 expression and translocation to the plasma membrane^68^. In this regard, it has been demonstrated that skeletal muscles of GLUT4-null mice exhibit a diminished glucose metabolism^69^ and increased fatigability^70^ accompanied of other metabolic disturbances such as growth retardation, abnormal adipose stores, cardiac hypertrophy and a shortened life span^70, 71^. Moreover, the conditional depletion of GLUT4 in mouse muscles^72^ or adipose tissue^73^ induces insulin resistance pointing to a critical role of GLUT4 in the glucose metabolism. Although HTZ mice did not show significant differences with their WT littermates in body weight, growth curves, organs mass or skeletal muscle glycogen^11^, we cannot rule out that the defects that we observed in GLUT4 trafficking (Fig. 6) could affect glucose homeostasis in the CNM condition. Therefore, future experiments are necessary to examine glucose uptake in isolated fibers of the HTZ KI-mice and its impacts on the body’s metabolism.

In summary, the current work shows that dynamin-2 is a key regulator of the actin dynamics in muscle cells and that dynamin-2 disfunction impacts on actin remodeling affecting intracellular trafficking. Our results argue for deregulation of these processes in dynamin-2-related CNM, which may contribute to the pathogenic mechanism of the disease.

## Methods

### Ethics

RCMH cell-line was established from a biopsy of a healthy human muscle (quadriceps) obtained from a trauma repairing surgery carried out at the Hospital Clínico Universidad de Chile, in compliance with national guidelines regarding the use of biopsy tissue for research. All experimental protocols were approved by the ethics committee of the Hospital Clínico Universidad de Chile and were conducted with the informed consent of the patient as described in the original report^27^.

All animal protocols described here were conducted in accordance with the approved protocols of the Bioethics Committee of the Facultad de Medicina of Universidad de Chile.

### Cell culture and transfection

RCMH myoblasts were cultured in poly-Lysine (Sigma, P4707) treated coverslips, mantained in DMEM F-12 medium (Gibco, 12400-024) supplemented with 10% fetal bovine serum (Gibco, 16000-044) and incubated at 37 °C in a 5% CO_2_ atmosphere. Transient transfections of GFP-GLUT4-HA, Dyn2WT-EGFP, Dyn2R369-EGFP, Dyn2R465W-EGFP, Dyn2R522H-EGFP or Dyn2K44A-GFP were performed using Lipofectamine 2000 (Invitrogen, 11668-019) according manufacturer’s instructions. Efficiently transfected cells were used 48 ± 2 h post-transfection. The DNA of the GFP-GLUT4-HA construct was kindly provided by Dr. Timothy McGrew (Cornell University).

### Dissection and isolation of Flexor Digitorium Brevis muscle fibers

The mouse-line KI-*Dnm2* ^R465W^ (HTZ) was established as previously reported^11^. Wild type (WT) and HTZ mice (C57BL/6 strain) were housed at room temperature with free access to food and water and maintained on light-darkness cycles of 12–12 h, according to standard protocols. Genotyping was performed by PCR as previously described^11^ using DNA extracted from tails. Primers used were: 3′-CTGCGAGAGGAGACCGAGC-5′ (forward) and 3′-GCTGAGCACTGGAGAGTGTATGG-5 (reverse). PCR products were electrophorated in agarose gels and bands of 445 bp and 533 bp represented WT and R465W mutated alleles, respectively.

Animals of 2–3 months of age were sacrificed by decapitation and used to isolate FDB muscles from hind legs. Freshly dissected muscles were incubated in a free calcium Tyrode solution (mM: 140 NaCl, 5 KCl, 10 Hepes/Na, 2 MgCl_2_, pH 7.2) containing 2.0 mg/ml of collagenase Type II (Worthington’s) during 2 h at 37 °C. Fibers were dissociated with tip-cutted micropipette tips in the presence of 10 µM of N-benzyl-p-toluen-sulphonamide (Sigma) to prevent fibers contraction.

### Immunofluorescence of isolated muscle fibers

To evaluate GLUT4 translocation, FDB fibers were stimulated by using 0.1 µM insulin in normal Tyrode solution (mM: 137 NaCl, 2.7 KCl, 1 MgCl_2_, 1.8 CaCl_2_, 0.2 Na_2_HPO_4_, 12 NaHCO_3_, 5.5 glucose, pH 7.2) during 15 min and then fixed during 15 min at room temperature with 4% paraformaldehyde (PFA). Fixed fibers were washed 3 times with PBS (mM: 137 NaCl, 2.7 KCl, 10 Na_2_HPO_4_, 2 KH_2_PO_4_, pH 7.4) plus 0.1% Triton X-100 (TX-100), permeabilized for 10 min with 0.5% TX-100 in PBS, blocked by 30 min with 5% of bovine serum albumin (BSA) in wash solution (PBS plus 0.1% TX-100) and then incubated overnight at 4 °C with a polyclonal antibody against GLUT4 (Abcam, ab654) in blocking solution (5% BSA in PBS, 0.1% TX-100). Images were acquired in a confocal microscope (upright Eclipse Nikon 80i) using an immersion-oil Plan Fluor 100x magnification objective (N.A 1.3) and identical exposure settings between compared samples. Single confocal images were acquired with the EZ-C1 (Nikon) software with a resolution of 1024 × 1024 pixels, analyzed and processed with the ImageJ software (NIH, USA). Expression of GLUT4 into the surface membrane was estimated by measuring the grayscale intensity value of GLUT4 in regions of interest (ROIs) along the sarcolemma (at both sides of fiber) averaged and normalized by the ROI area.

To evaluate overall actin organization pattern, dissociated fibers were fixed, permeabilized, and incubated during 40 min with 1 µM of Phalloidin-Rhodamine-B (Sigma, P1951). Fibers were incubated with DAPI to stain myonuclei and visualized by confocal microscopy.

### F/G actin assay

RCMH myoblasts, plated in 100 mm dishs at a density of 1 × 10^7^ cells, were treated with: 2 µM jasplakinolide (Invitrogen, J7473), 4 µM cytochalasine D (Gibco,PHZ1063), 20 µM dynole 34-2 (Abcam, ab120463), 20 µM dynole 31-2 (Abcam, ab120464) or DMSO during 15 min at 37 °C and then lysed in conditions that stabilize filamentous (F-) and monomeric (G-) actin using an F/G actin *in vivo* commercial assay (Cytoskeleton Inc., BK037). Lysated extracts were ultracentrifuged at 100,000 g for 1 h at 37 °C in order to separate precipitated (F-actin) and soluble (G-actin) fractions. F-actin pellet was resuspended in a depolymerizing buffer (Cytoskeleton Inc., BK037) and then F-and G-actin fractions were diluted in loading buffer (50 mM Tris–HCl, 2% SDS, 10% glycerol, 1% beta-mercaptoethanol and bromophenol blue). Samples were subjected to electrophoresis in 12%-SDS-PAGE, transfered to a PVDF membrane, blocked for 1 h with 5% non-fat milk in TBS-T (mM: 150 NaCl, 50 Tris/HCl, pH 7.4, 0.05% Tween-20), incubated with a polyclonal antibody against actin (1:500; Cytoskeleton Inc., BK037) and developed by chemiluminiscence. *Tibialis Anterior* muscles freshly dissected from WT and HTZ mice were lysed and processed using the same protocol. Densitometric analysis of F- and G-actin bands was performed with the ImageJ software and the F/G ratio was determined.

### Actin polymerization assay

Cultured RCMH myoblasts were permeabilized during 6 min with 20 μM digitonin in KGEP buffer (mM: 139 K-glutamate, 20 PIPES, 5 EGTA, 2 ATP-Mg^2+^ and 10 µM of free Ca^2+^, pH 6.6) in the presence of pharmacological agents (100 µM dynasore, 20 µM dynole 34-2, 20 µM dynole 31-2, 4 µM CytoD or DMSO) and 0.3 µM G-actin-AF488 (Molecular Probes, A12373). When it is specified, RCMH myoblasts transfected with dynamin-2 EGFP constructs were permeabilized with digitonin in KGEP buffer in the presence of G-actin-AF568 (Molecular Probes, A12374) at least 48 h post-transfection. Then, cells were fixed during 15 min with 4% PFA, DAPI-incubated for nuclei staining and visualized by confocal microscopy. Single Z-images were captured at the equatorial plane of cells and 1024 × 1024 pixels images were analyzed and processed using the ImageJ software. The amount of new actin filaments formed was quantified by drawing the cell periphery and measuring the grayscale integrated density fluorescence of AF488 (or AF568) after background subtraction, and normalized by the cell area.

The same assay was performed in FDB fibers isolated from WT and HTZ mice, but in this case *de novo* actin polymerization was induced by insulin stimulation (0.1 µM during 10 min). Then, fibers were permeabilized with digitonin in KGEP buffer in the presence of 0.5 µM G-actin-AF488 conjugate, fixed and visualized by confocal microscopy using identical exposure settings between compared samples. Images were acquired at the fiber bottom and newly assembled actin was quantified in the raw images as the grayscale integrated density fluorescence of AF488 after background subtraction, and normalized by the fiber area.

### GLUT4 translocation assay in RCMH cells

In order to quantify GLUT4 insertion into the plasma membrane, transfected RCMH myoblasts, expressing the construct GFP-GLUT4-HA, were stimulated for 5 min at 37 °C with 20 µM of ionomycin in normal Tyrode solution in the presence of 20 µM dynole 34-2, 20 µM dynole 31-2, 4 µM CytoD or DMSO and fixed with 4% PFA during 15 min at room temperature. To stain the exofacial HA-epitope, fixed cells were immunolabeled with an anti-HA antibody (Invitrogen, 715500) without permeabilization, developed with a Cy3-conjugated secondary antibody (Jackson Immuno Research,111-165-047) and visualized by TIRF microscopy (Nikon Eclipse Ti-E), using a 60×/1.49 NA Plan APO TIRF objective (Nikon). Surface Reflective Interference Contrast (SRIC; NIKON) was used to identify the areas of membrane contact with the glass surface. 16-bit images of 960 × 720 pixels resolution were acquired with the NIS-Element viewer 4.3 software. Using the ImageJ software, raw images were converted to 8-bit, and plasma membrane insertion of GFP-GLUT4-HA was quantified as a ratio between HA (Cy3) and GFP fluorescence intensities in the evanescent field, after background subtraction.

Plasma membrane insertion of endogenous GLUT4 was assessed in myoblasts expressing dynamin-2-EGFP constructs. Transfected RCMH cells were stimulated with 20 µM ionomycin for 5 min, fixed, immunolabeled with a monoclonal anti-GLUT4 antibody (Abcam, ab35826) and visualized by TIRF microscopy. 16-bit raw images were converted to 8-bit, using the ImageJ software and plasma membrane insertion of endogenous GLUT4 was quantified as the total fluorescence intensity of GLUT4 in the evanescent field after background subtraction.

### Surface membrane biotinylation

FDB muscles were dissected from WT and HTZ mice, stabilized during 1 h at room temperature in Tyrode solution, bubbled with a mixture of 5% CO_2_ and 95% O_2_, and *ex vivo* stimulated with 0.1 µM insulin during 30 min. Then, muscles were exposed to 1 mg/ml of EZ link Sulfo-NHS-SS-biotin (Thermo Scientific #21331) in Tyrode solution at 4 °C for 60 min. Biotinylation was quenched with 100 mM of cold glycine in Tyrode solution; muscles were washed three times with ice-cold Tyrode, frozen and pulverized in liquid nitrogen and then lysed in ice-cold lysis buffer (mM: 1 EDTA, 50 NaCl, 20 TrisHCl, pH 7.5 plus 1% TX-100 and 0.1% SDS). Homogenates were centrifuged at 14.000 g for 10 min and supernatants were mixed with 50 µl of streptavidin-agarose beads (Thermo Scientific #29204) overnight at 4 °C with gentle agitation and then centrifuged at 14.000 g for 3 min. Biotinylated and non-biotinylated fractions were diluted in loading buffer, subjected to SDS-PAGE and western blot to evaluate GLUT4 and GAPDH expression using specific antibodies (rabbit-polyclonal-anti GLUT4, Abcam ab654 and mouse-monoclonal-anti-GAPDH, Abcam ab9484, respectively).

### GLUT4 immunolabeling in human skeletal muscle biopsies

Human open muscle biopsies from two patients carrying the CNM- dynamin-2 mutation p.R465W, one patient carrying the CNM-dynamin-2 mutation p.R369Q, one patient with dystrophy harboring a mutation in dysferlin (dysferlinopathy), one patient with dystrophy harboring a mutation in dystrophin (dystrophinopathy) and one healthy control muscle were performed at the Centre de Référence de Pathologie Neuromusculaire Paris-Est, Institut de Myologie, GHU Pitié-Salpêtrière, Assistance Publique-Hôpitaux de Paris, GH Pitié-Salpêtrière, Paris, France, following written informed consent specially dedicated for diagnosis and research. All experimental protocols were approved by the ethics committee of the Centre de Référence de Pathologie Neuromusculaire Paris-Est, Institut de Myologie, GHU Pitié-Salpêtrière, Assistance Publique-Hôpitaux de Paris, GH Pitié-Salpêtrière, Paris, France. For conventional histochemical and immunocytochemical techniques see reference^74^. Briefly, frozen muscle samples were stained with a specific anti-GLUT4 antibody and revealed by immunoperoxidase techniques (Roche-Benchmark). Digital photographs were obteined with a Zeiss AxioCam HRc linked to a Zeiss Axioplan Bright Field Microscope (Zeiss, Germany).

### Statistics

Data are expressed as mean ± standard error of the mean (SEM); in the figure captions “n” refers to the number of tested RCMH myoblasts from at least three different cultures or the number of muscle fibers from at least three different animals per genotype. The normality of all raw data was evaluated automatically with the GraphPad InStat sofware. Statistical comparisons were performed utilizing a two-tail Welch’s corrected t-test for parametric data or a two-tail Mann-Whitney test for non-parametric data.

## Electronic supplementary material

Supplementary Info
