# Supplementary material for: Dynamin-2 mutations linked to Centronuclear Myopathy impair actin-dependent trafficking in muscle cells
Source: Sci Rep. 2017 Jul 4;7:4580. doi: 10.1038/s41598-017-04418-w (PMC5496902; doi:10.1038/s41598-017-04418-w)
Supplement: Supplementary file 1 — Supplementary Info [file 41598_2017_4418_MOESM1_ESM.pdf]

Dynamin-2 mutations linked to Centronuclear Myopathy impair actin-dependent trafficking in muscle cells.

Arlek M. González-Jamett[1,6,*], Ximena Baez-Matus[1], María José Olivares[1], Fernando Hinostroza[1,+], Maria José Guerra-Fernández[1], Jacqueline Vasquez-Navarrete[1], Mai Thao Bui[2], Pascale Guicheney[3], Norma Beatriz Romero[2], Jorge A. Bevilacqua[4], Marc Bitoun[5], Pablo Caviedes[6, †], Ana M. Cárdenas[1]

[1] Centro Interdisciplinario de Neurociencia de Valparaíso. Facultad de Ciencias. Universidad de Valparaíso, Valparaíso, Chile.

[2] Université Sorbonne, UPMC Univ Paris 06, INSERM UMRS974, CNRS FRE3617, Center for Research in Myology, and Centre de référence de Pathologie Neuromusculaire Paris-Est, Institut de Myologie, GHU Pitié-Salpêtrière, Assistance Publique-Hôpitaux de Paris, GH Pitié-Salpêtrière, Paris, France

[3] INSERM, UMR_S1166, Paris, France, Sorbonne Universités, UPMC Univ Paris 06, UMR_S1166, Institute of Cardiometabolism and Nutrition (ICAN), Paris, France.

[4] Programa de Anatomía y Biología del Desarrollo, ICBM, Facultad de Medicina, Departamento de Neurología y Neurocirugía, Hospital Clínico Universidad de Chile, Universidad de Chile, Santiago, Chile.

[5] Research Center for Myology, UPMC Univ Paris 06 and INSERM UMRS 974, Institute of Myology, Paris, France.

[6] Programa de Farmacología Molecular y Clinica, ICBM, Facultad de Medicina, Universidad de Chile, Santiago, Chile.

[+] F.H is a PhD student of the program Doctorado en Ciencias, mención Neurociencia, Universidad de Valparaíso.

[†]Address all correspondence regarding the use of the RCMH cell line to Pablo Caviedes (pcaviede@med.uchile.cl)

* correspondence should be addressed to AG-J: arlek.gonzalez@cinv.cl

SUPPLEMENTARY FIGURE LEGENDS

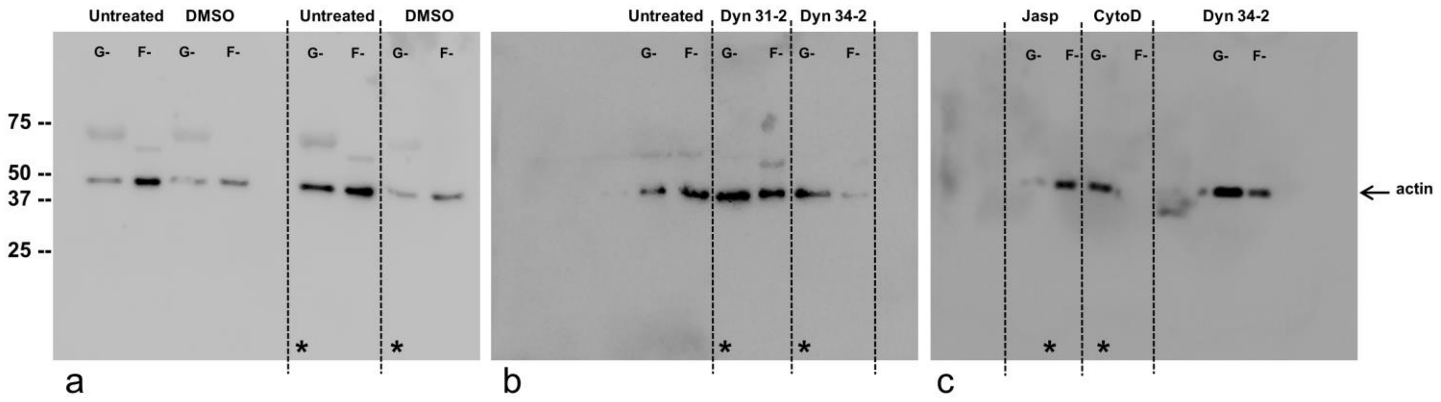

Supplementary figure S1. Full length uncropped images of the western blots used in Fig.1c. These are three different western blots from F/G-actin samples of RCMH lysates. Although not necessarily at the same time, all gels have been run under the same experimental conditions. Western-blots were developed with a specific anti-actin antibody (Cytoskeleton, Inc, BK037). **(a)** Gel in which untreated and DMSO-treated lysates were run, **(b)** gel in which untreated lysates and treated with dynole 31-2 (Dyn31-2) and dynole 34-2 (Dyn34-2) lysates were run, **(c)** gel in which lysates treated with jasplakinolide (Jasp), cytochalasine (CytoD) and Dynole34-2 were run. Dotted lines and * symbols delimit each cropped blot used in Fig.1c. Molecular weights (kDa) are marked at left.

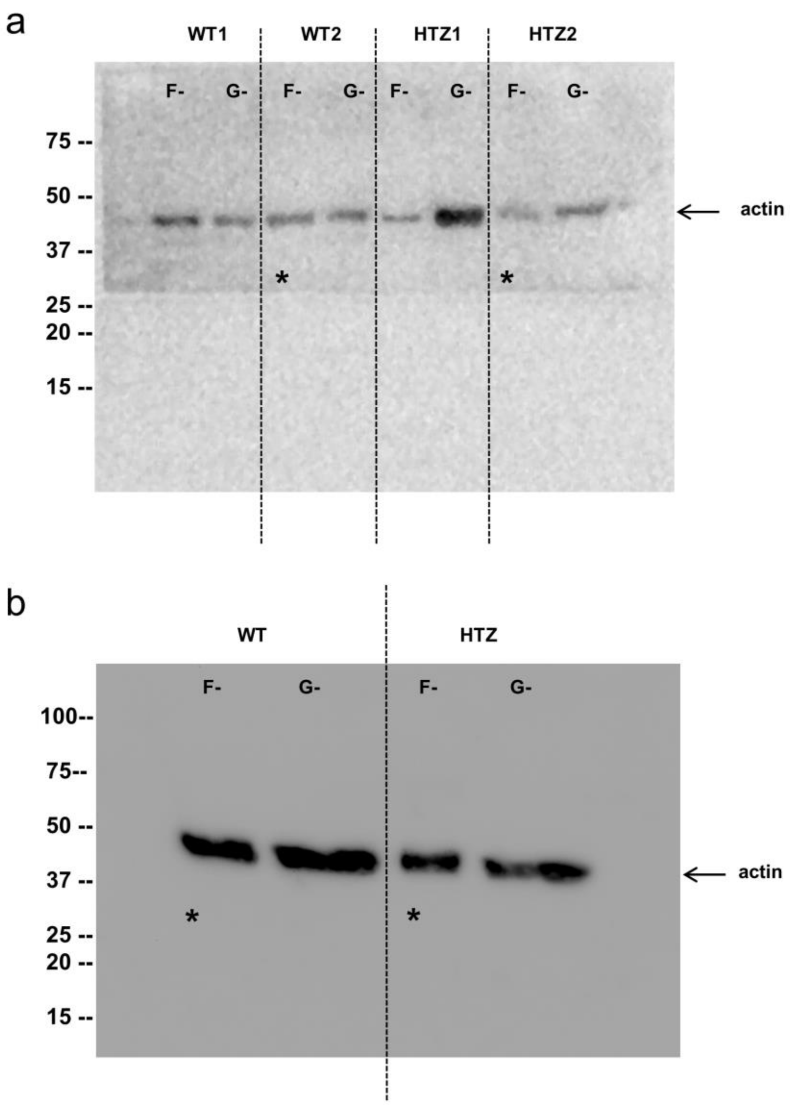

Supplementary figure S2. (a-b) Full length uncropped images of the blots used in Fig.5b. F/G actin samples from *tibialis anterior* (a) and FDB muscles (b) of WT and HTZ animals. Dotted lines and * symbols delimit the cropped blots used in Fig.5b. Molecular weights (kDa) are marked at left. Both membranes were cut. The membrane in (a) was stained in top with the anti-actin antibody (Cytoskeleton, Inc, BK037) and in bottom with an anti-Cav-3 antibody (Santa Cruz, sc-5310). This latter gave no bands. The membrane in (b) was only stained with the anti-actin antibody.

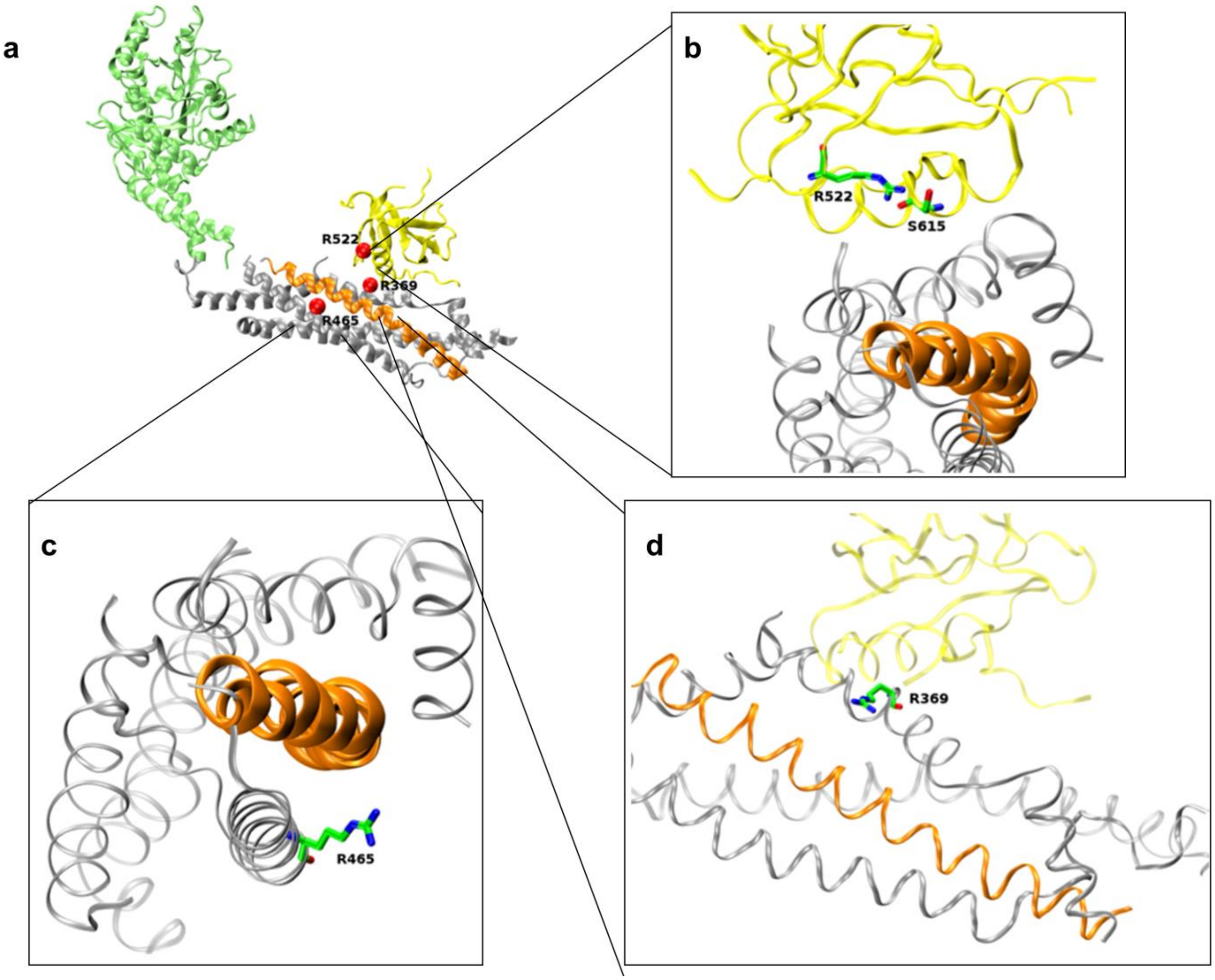

Supplementary figure S3. (a) Crystallographic structure of human dynamin-1 (PDB ID: 3SNH). In orange is the putative actin-binding motif comprising residues 399 to 444. In red are highlighted arginine residues R369, R465 and R522 that are substituted by tryptophan (R369W and R465W) and histidine (R522H) in CNM-mutations. **(b)** R522 residue localizes in the PH domain of dynamin and appears far from the putative actin binding motif. **(c)** R465 residue localizes in the middle domain of dynamin, near to the alpha-helix that theoretically interacts with actin filaments. **(d)** R369 residue is also located in the middle domain of dynamin near to the actin-binding motif and also interacts with residues in the PH domain. Dynamin GTP-ase domain is shown in green, middle domain in gray and PH domain in yellow.

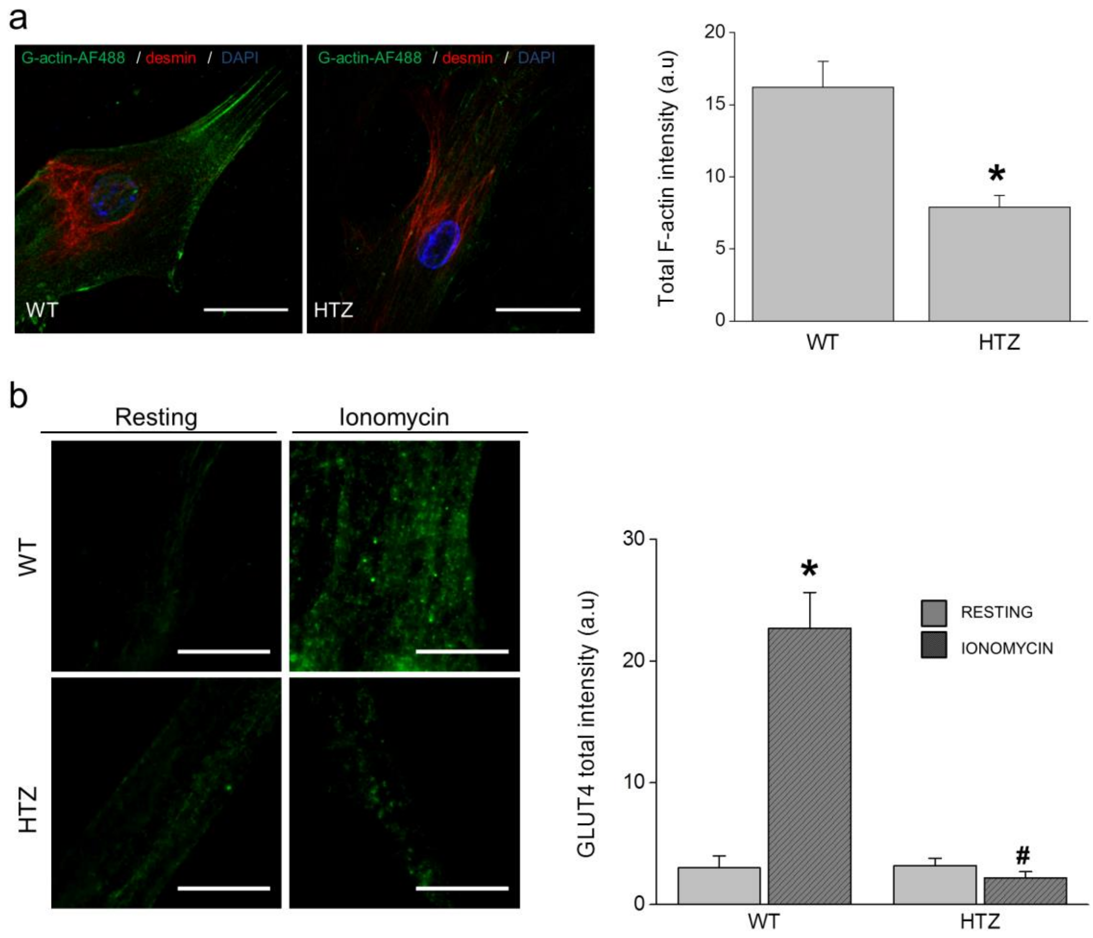

Supplementary figure S4. Myoblasts were isolated from *tibialis anterior* muscles of WT and HTZ mice, cultured in poly-Lysine (Sigma, P4707) treated coverslips, maintained in DMEM F-12 medium (Gibco, 12400-024) supplemented with 10% fetal bovine serum (Gibco, 16000-044) and incubated at 37°C in a 5% CO_2 atmosphere until experimentation. **(a)** *De novo* actin polymerization (green) was reduced in HTZ myoblasts compared to WT myoblasts. An anti-desmin antibody (Abcam, ab32362) was used as a myoblast marker (red). Scale bar = 10 µm; data are mean total F-actin intensity ± SEM; N is between 33 and 35 cells from at least 4 different animals per genotype; *p<0.05 respect to WT cells. **(b)** Endogenous GLUT4 translocation is reduced in HTZ myoblasts compared to WT myoblasts upon ionomycin stimulation. Scale bar = 10 µm; data are mean GLUT4 intensity ± SEM; N is between 8 and 14 cells from three different animals per genotype.

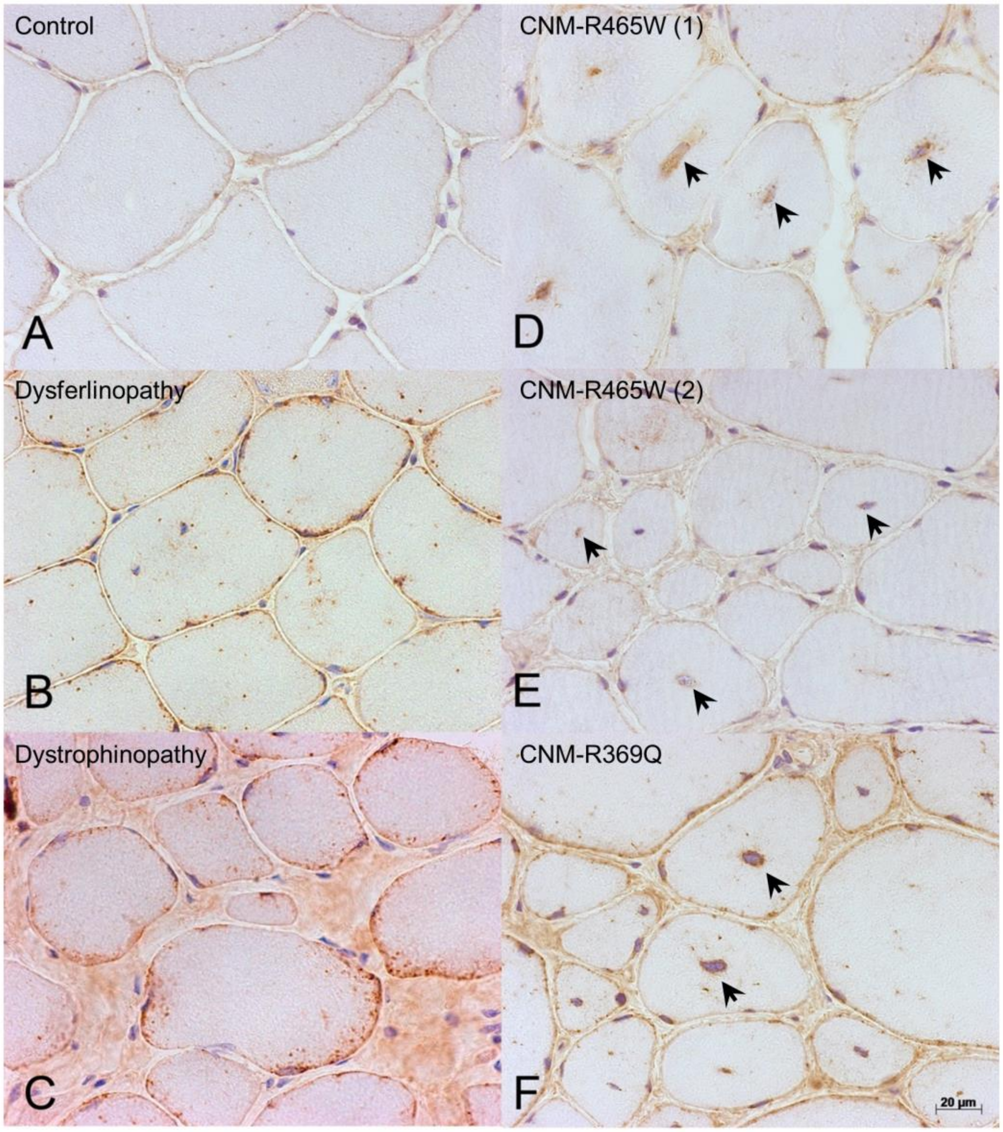

Supplementary figure S5. Skeletal muscle biopsies from a healthy patient (A), a dysferlinopathy patient (B), a dystrophinopathy patient (C), two CNM- patients harboring the p.R465W mutation (D-E) and one CNM-patient carrying the p.R369Q mutation (F) were stained with a specific anti-GLUT4 antibody and revealed by immunoperoxidase techniques (Roche-Benchmark). Digital photographs were obteined with a Zeiss AxioCam HRc linked to a Zeiss Axioplan Bright Field Microscope (Zeiss, Germany). Note that GLUT4 accumulates around centralized nuclei in biopsies from CNM-patients (D-F) but it is distributed similar to the control (A) in dystrophic biopsies (B-C). Scale bar is 20 µm.

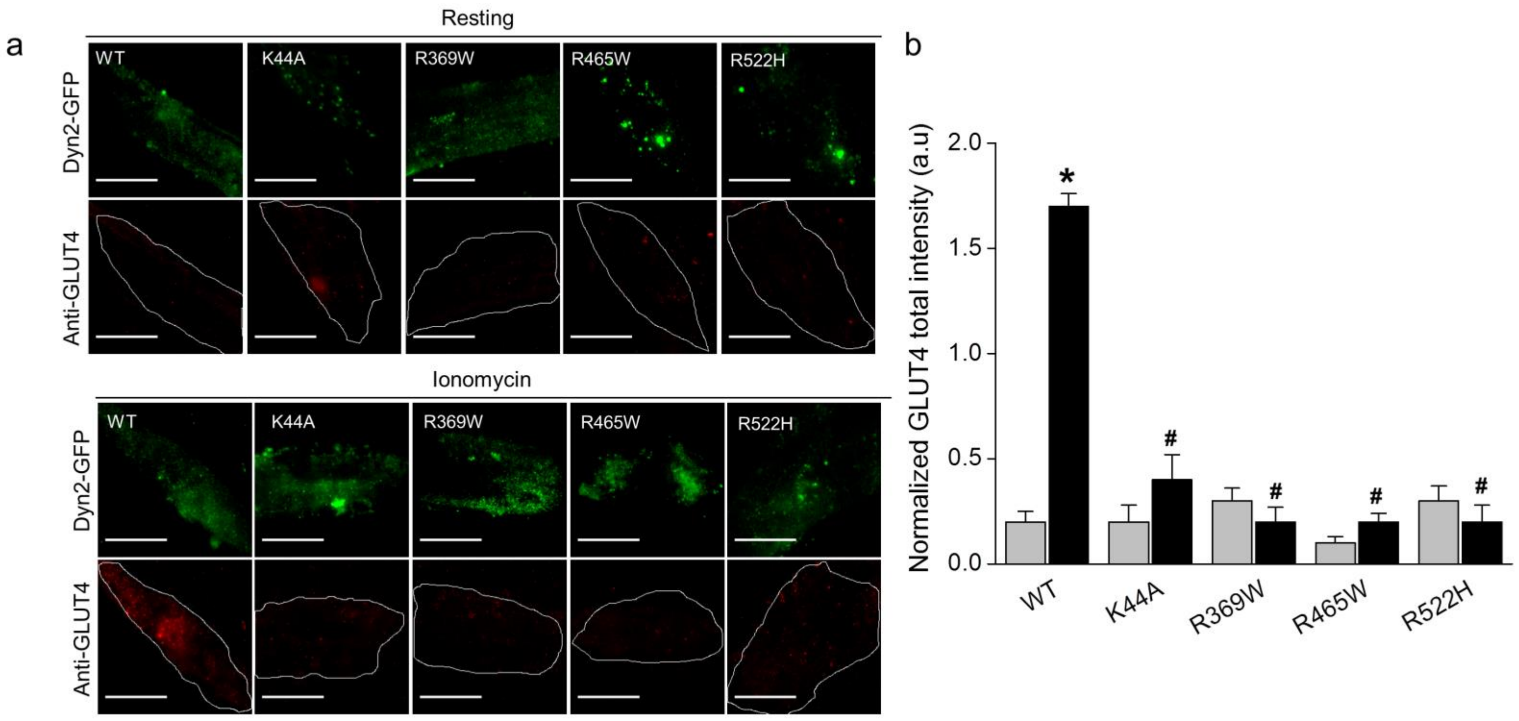

Supplementary figure S6. (a-b) RCMH cells efficiently transfected with EGFP-dynamin-2 constructs were stimulated with ionomycin. GLUT4 translocation to the plasma membrane was evaluated in TIRFM and total EGFP signal was estimated in epifluorescence using identical acquisition and analysis settings. All images were acquired using a TIRF microscopy (Nikon Eclipse Ti-E) with a 60X objective, numerical aperture of 1.49 and laser excitation power of 20 mW for the laser 488 and 1.5 mW for the laser 543. In all cases (epifluorescence and TIRF) 16-bit images of 960x720 pixels resolution were acquired (without selecting specific regions) using the NIS-Element Viewer 4.3 software. Raw images were analyzed using the ImageJ software. 16-bit images were converted to 8-bit and background was subtracted from a 2x2 pixel region outside cells, subtracting two standard deviations of background from the mean fluorescence intensity. ROIs were defined by drawing the outline of cells (white lines in the bottom panels) and mean fluorescence intensity was obtained by dividing the integrated signal for the respective ROI

area. **(a)** Representative images at resting and ionomycin-stimulated conditions. Scale bar= 10 µm. **(b)** Data are mean ± SEM; * $p < 0.05$ respect to the resting condition; # $p < 0.05$ respect to ionomycin-stimulated cells transfected with the WT construct. N is between 6 and 15 cells from three different cultures.
